# Dissemination of Verona Integron-encoded Metallo-β-lactamase among clinical and environmental Enterobacteriaceae isolates in Ontario, Canada

**DOI:** 10.1038/s41598-020-75247-7

**Published:** 2020-10-29

**Authors:** Philipp Kohler, Nathalie Tijet, Hyunjin C. Kim, Jennie Johnstone, Tom Edge, Samir N. Patel, Christine Seah, Barbara Willey, Brenda Coleman, Karen Green, Irene Armstrong, Kevin Katz, Matthew P. Muller, Jeff Powis, Susan M. Poutanen, David Richardson, Alicia Sarabia, Andrew Simor, Allison McGeer, Roberto G. Melano, The Toronto Invasive Bacterial Diseases Network (TIBDN), Agron Plevneshi, Wallis Rudnick, Frances Jamieson, Barbara Yaffe, Larissa Matukas, James Downey, Wayne Gold, Sharon Walmsley, Mahin Baqi, Mary Vearncombe, Abdelbaset Belhaj, Ian Kitai, Danny Chen, Eileen de Villa, Hani Dick, Nataly Farshait, King S. Lee, Sigmund Krajden, Michael Lingley, Reena Lovinsky, David Rose, Sharon O’Grady, Anne Opavsky, Krystyna Ostrowska, Astrid Petrich, Susan Richardson, Neil Rau, Daniel Ricciuto, Valerie Sales, Deborah Yamamura

**Affiliations:** 1 Sinai Health System, Toronto, ON Canada; 2 Public Health Ontario Laboratory, Toronto, ON Canada; 3 University of Toronto, Toronto, ON Canada; 4 Environment and Climate Change Canada, Burlington, ON Canada; 5 McMaster University, Hamilton, ON Canada; 6 Toronto Public Health, Toronto, ON Canada; 7 North York General Hospital, Toronto, ON Canada; 8 St. Michael’s Hospital, Toronto, ON Canada; 9 Michael Garron Hospital, Toronto, ON Canada; 10 University Health Network, Toronto, ON Canada; 11 William Osler Health System, Brampton, ON Canada; 12 Trillium Health Partners, Toronto, ON Canada; 13 Sunnybrook Health Sciences Centre, Toronto, ON Canada; 14 Rouge Valley Health System, Toronto, ON Canada; 15 Mackenzie Health, Richmond Hill, ON Canada; 16 Region of Peel Public Health, Brampton, ON Canada; 17 Vita-Tech Canada Inc., Markham, ON Canada; 18 Humber River Regional Hospital, Toronto, ON Canada; 19 St. Joseph’s Health Centre, Toronto, ON Canada; 20 Southlake Regional Health Centre, Newmarket, ON Canada; 21 The Scarborough Hospital, Toronto, ON Canada; 22 Bridgepoint Sinai Health System, Toronto, ON Canada; 23 Joseph Brant Memorial Hospital, Burlington, ON Canada; 24 Trillium Health Partners, Mississauga, ON Canada; 25 The Hospital for Sick Children, Toronto, ON Canada; 26 Halton Healthcare, Oakville, ON Canada; 27 Lakeridge Health, Oshawa, ON Canada; 28 Markham Stouffville Hospital, Markham, ON Canada; 29 Hamilton Health Sciences Centre, Hamilton, ON Canada

**Keywords:** Epidemiology, Clinical microbiology

## Abstract

Surveillance data from Southern Ontario show that a majority of Verona Integron-encoded Metallo-β-lactamase (VIM)-producing *Enterobacteriaceae* are locally acquired. To better understand the local epidemiology, we analysed clinical and environmental *bla*_VIM_-positive *Enterobacteriaceae* from the area. Clinical samples were collected within the Toronto Invasive Bacterial Diseases Network (2010–2016); environmental water samples were collected in 2015. We gathered patient information on place of residence and hospital admissions prior to the diagnosis. Patients with and without plausible source of acquisition were compared regarding risk exposures. Microbiological isolates underwent whole-genome sequencing (WGS); *bla*_VIM_ carrying plasmids were characterized. We identified 15 patients, thereof 11 with *bla*_VIM-1_-positive *Enterobacter hormaechei* within two genetic clusters based on WGS. Whereas no obvious epidemiologic link was identified among cluster I patients, those in cluster II were connected to a hospital outbreak. Except for patients with probable acquisition abroad, we did not identify any further risk exposures. Two *bla*_VIM-1_-positive *E. hormaechei* from environmental waters matched with the clinical clusters; plasmid sequencing suggested a common ancestor plasmid for the two clusters. These data show that both clonal spread and horizontal gene transfer are drivers of the dissemination of *bla*_VIM-1_-carrying *Enterobacter hormaechei* in hospitals and the aquatic environment in Southern Ontario, Canada.

## Introduction

The global dissemination of metallo-β-lactamases (MBLs) constitutes a severe threat to modern healthcare. MBLs exhibit a broad hydrolytic spectrum, which inactivates many currently used β-lactams. The Verona Integron-encoded Metallo-β-lactamase (VIM) is among the most common MBLs causing human infection^1^. Over the last decade this carbapenemase has become a serious health threat in healthcare institutions in countries such as Greece, Italy, and Spain^2–4^. It has been associated with outbreaks of hospital acquired infections due to *Pseudomonas aeruginosa* and *Enterobacteriaceae*, and has been found in both sewage and surface water in many countries^5–10^. The successful dissemination of the *bla*_VIM_-gene can be explained by its location on gene cassettes of class 1 integrons which themselves are usually found on mobile genetic elements such as transposons and plasmids^1,11^.

In North America, VIM-producing *Enterobacteriaceae* have been found in numerous geographic areas but are less common than carbapenemase-producing *Enterobacteriaceae* (CPE) carrying other carbapenemases^12–15^. In southern Ontario, population-based surveillance from 2007 to 2015 showed that the New Delhi Metallo-β-lactamase (NDM) and the *Klebsiella pneumoniae* Carbapenemase (KPC) were the most commonly detected carbapenemases (56% and 25%, respectively), whereas the VIM carbapenemase only accounted for 5% of cases. In contrast to patients with other CPE, two-thirds of patients with *bla*_VIM_-positive *Enterobacteriaceae* did not have a history of hospital admission or travel abroad, suggesting local acquisition^16^. This prompted us to study the molecular epidemiology of clinical VIM isolates, to compare these to environmental water isolates from our area, and to search for risk factors for VIM acquisition.

## Results

### Clinical and environmental isolates

Between 2007 and January 2016, Toronto Invasive Bacterial Diseases Network (TIBDN) surveillance identified 300 patients colonized or infected with CPE, thereof 15 unique patients (5%) colonized or infected by 16 non-duplicate *bla*_VIM_-positive *Enterobacteriaceae*. Eleven patients carried *E. cloacae* complex isolates, one patient each carried *E. coli*, *Citrobacter freundii,* and *Klebsiella pneumoniae* and one patient carried both *C. freundii* and *K. pneumoniae*. All isolates were positive for *bla*_VIM-1_, except for one *C. freundii* carrying *bla*_VIM-2_. Two patients were co-colonized with other CPEs: one with an NDM-producing *E. coli* and an NDM/OXA-48 co-producing *K. pneumoniae*, and one with an NDM-producing *K. pneumoniae.* Environmental water sampling in 2015 detected four *bla*_VIM-1_-positive isolates belonging to the *E. cloacae* complex, three from surface water (1 site) and one from sewage water (1 site). After submission, the whole genome sequences (WGS) of all fifteen of the *E. cloacae* complex isolates were 98.412% to 99.928% identical by average nucleotide identity to the type genome of *E. hormaechei*, with 75.6% to 89.4% coverage of the genome, according to NCBI analysis using average nucleotide identity (ANI) and comparing the submitted genome sequences against the genomes of the type strains that are already in GenBank. Therefore, we are using *E. hormaechei* as the organism name for these isolates.

### MLST and SNP analysis

**Figure 1:**
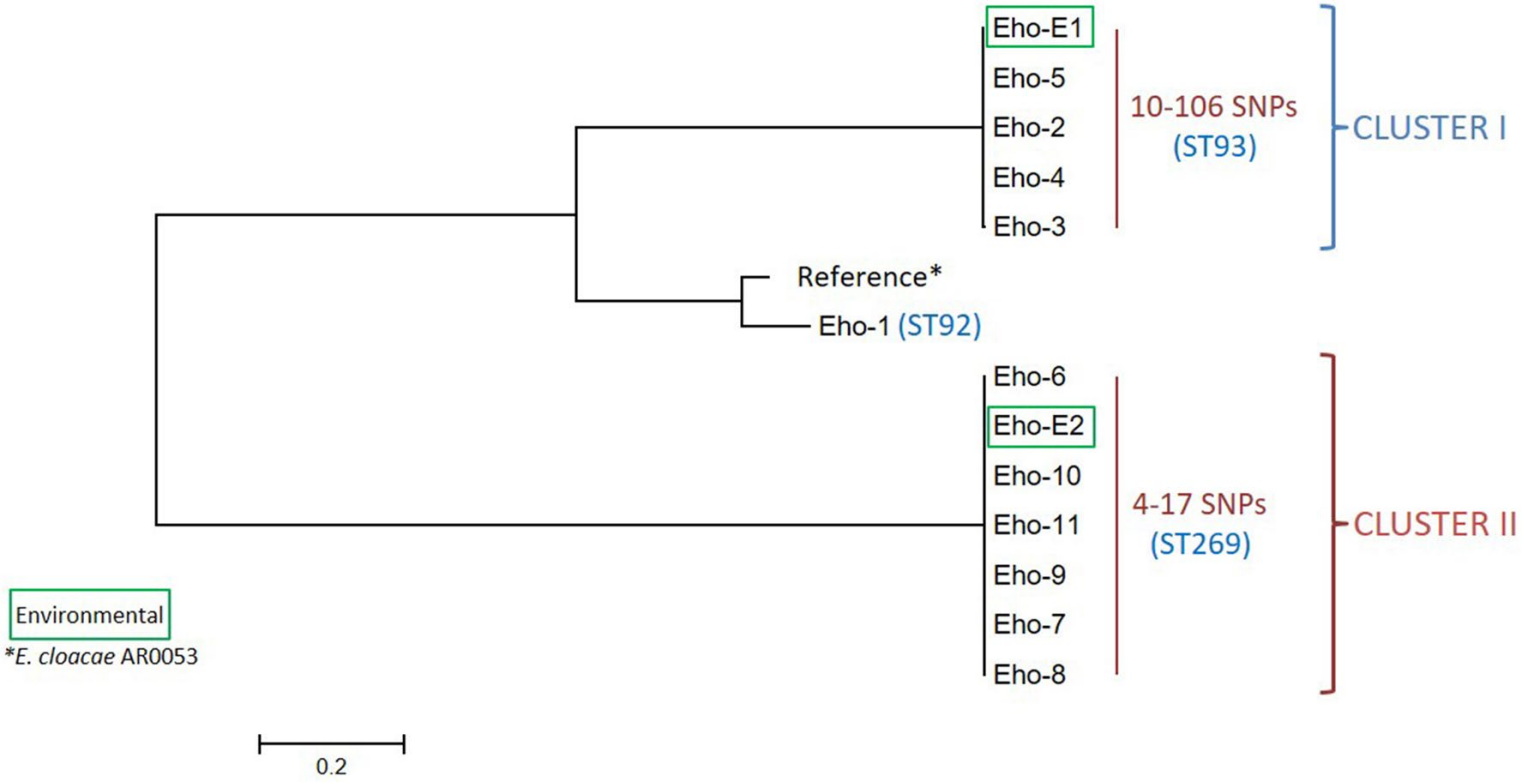
Single nucleotide polymorphisms (SNP) analysis for *bla*_VIM-1_-carrying *Enterobacter hormaechei.*

SNP analysis showed two patient clusters among *E. hormaechei*. The four patients with isolates comprising cluster I were identified between 2010 and 2012 (10 to 106 SNPs, all ST93). The six patients with isolates comprising cluster II were identified between 2014 and 2016 (4 to 17 SNPs, all ST269) (Table 1, Fig. 1). Almost 80,000 SNPs of difference were found between clusters. One isolate not belonging to either cluster was typed as ST92 (~ 50,000 and ~ 76,000 SNPs of difference with cluster I and II, respectively). Three environmental *E. hormaechei* from surface water, belonging to ST93, displayed 39 to 106 SNPs of difference with the clinical isolates in cluster I. The environmental sample from sewage water was ST269, showing 4 to 13 SNPs of difference with the clinical ST269 isolates in cluster II (Fig. 1). One *C. freundii* was identified as ST129, whereas the other exhibited a new, not yet assigned ST (2,024 SNPs of difference between them). The *E. coli* isolate was of ST131 and the two *K. pneumoniae* were of ST376 and ST17, respectively (22,961 SNPs of difference) (Table 1).

**Table 1:** Characteristics of 16 non-duplicate *bla*_VIM_-positive clinical *Enterobacteriaceae* and four related environmental isolates.

Patient	Species	Source	Date	Travel	Epi Risk*	Enzyme	Isolate	ST^†^
1	*E. hormaechei*	Rectal	Sep-12	Croatia (H)	Yes	VIM-1	Eho-1	92
2	*E. hormaechei*	Urine	Jul-11	None	No	VIM-1	Eho-2^‡^	93
3	*E. hormaechei*	Urine	Aug-11	None	No	VIM-1	Eho-3^‡^	93
4	*E. hormaechei*	Urine	Feb-12	None	No	VIM-1	Eho-4^‡^	93
5	*E. hormaechei*	Urine	Jun-12	None	No	VIM-1	Eho-5^‡^	93
6	*E. hormaechei*	Rectal	Feb-14	None	No	VIM-1	Eho-6^§^	269
7	*E. hormaechei*	Urine	Jul-14	None	Yes (OB)^&^	VIM-1	Eho-7^§^	269
8	*E. hormaechei*	Dialysis fluid	Jun-15	None	Yes (OB)	VIM-1	Eho-8^§^	269
9	*E. hormaechei*	Dialysis fluid	Sep-15	None	Yes (OB)	VIM-1	Eho-9^§^	269
10	*E. hormaechei*	Rectal	Jan-16	None	Yes (OB)	VIM-1	Eho-10^§^	269
11	*E. hormaechei*	Rectal	Jan-16	None	Yes (OB)	VIM-1	Eho-11^§^	269
12	*C. freundii*	Sputum	Dec-12	Portugal (H)	Yes	VIM-2	Cfr-12	129
13a	*C. freundii*	Rectal	Dec-15	None	No	VIM-1	Cfr-13a	New
13b	*K. pneumoniae*	Rectal	Dec-15			VIM-1	Kpn-13b	17
14	*K. pneumoniae*	Rectal	Sep-14	Egypt (H)	Yes	VIM-1	Kpn-14	376
15	*E. coli*	Rectal	Nov-15	Europe	No	VIM-1	Eco-15	131
E1	*E. hormaechei*	Environment	Aug-15	NA	NA	VIM-1	Eho-E1^‡^	93
E2	*E. hormaechei*	Environment	Aug-15	NA	NA	VIM-1	Eho-E2^§^	269
E3	*E. hormaechei*	Environment	Aug-15	NA	NA	VIM-1	Eho-E3	93
E4	*E. hormaechei*	Environment	Aug-15	NA	NA	VIM-1	Eho-E4	93

*H* hospital, *Epi* epidemiologic, *ST* sequence type, *VIM* Verona integron-encoded metallo-β-lactamase, *OB* outbreak.

*Hospital admission outside of North America OR spatiotemporal exposure to patient with highly-related isolate.

^†^ST, Sequence types, determined by multilocus sequence typing.

^&^Hospital outbreak with patient 6 as first case identified, and patients #7–11 exposed to contaminated shower drains on same hospital unit (17).

^‡^Cluster I.

^§^Cluster II.

### Epidemiological factors associated with VIM-acquisition without known risk factors

**Figure 2:**
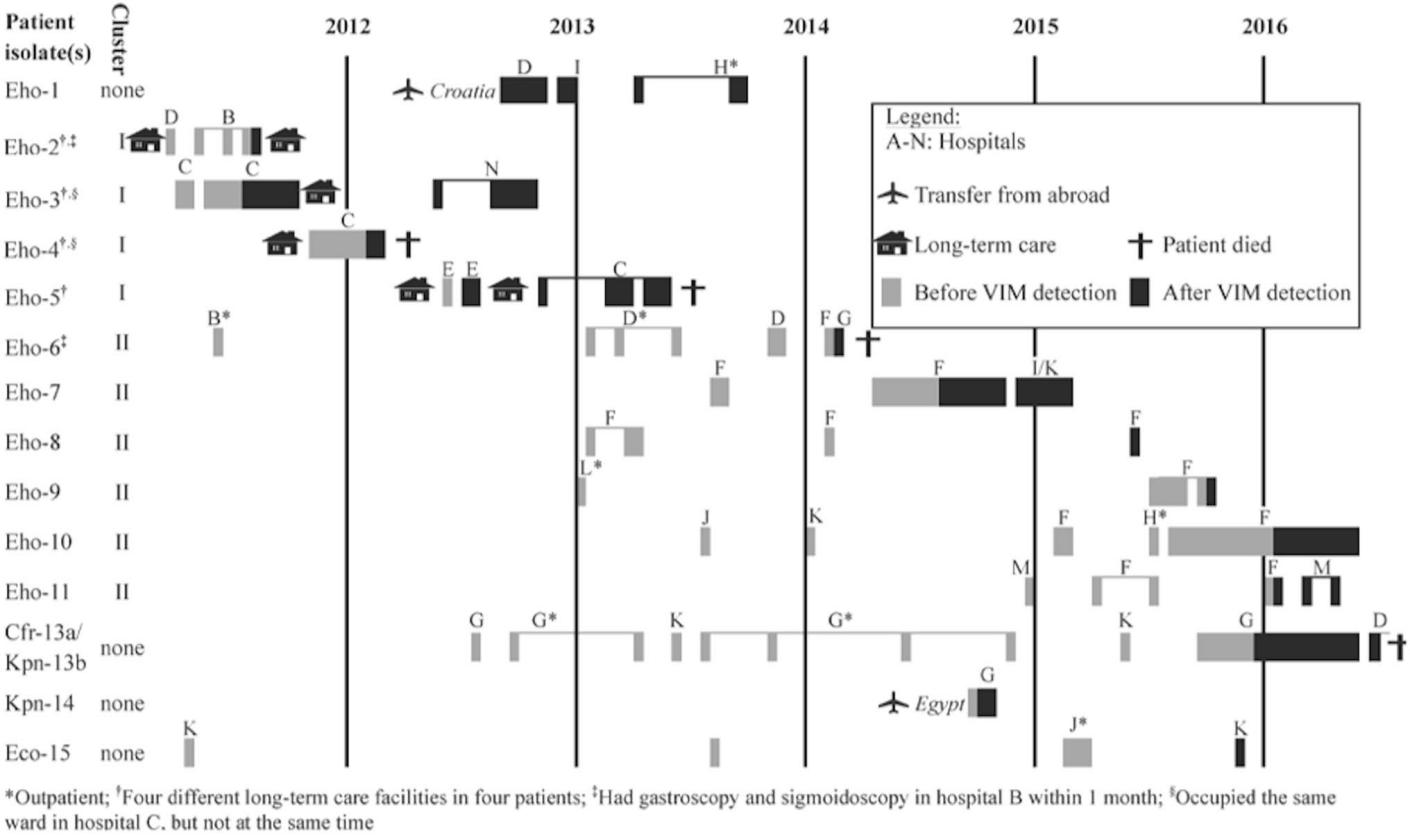
Inpatient and outpatient healthcare contacts in the Toronto Invasive Bacterial Diseases Network surveillance area as well as hospital admissions abroad of patients with *bla*_VIM-1_-positive *Enterobacteriaceae.* Each black or grey bar represents a hospital visit or admission with the different hospitals represented by different letters, and out-patient visits only marked with an asterisk.

Among the 15 patients, three (including the patient with *bla*_VIM-2_ positive *C. freundii*) had a healthcare visit abroad (Croatia, Egypt, and Portugal, respectively) and one reported travel outside North America (to Austria, Germany, and France) without healthcare contact (Table 1). For all 14 patients with *bla*_VIM-1_, inpatient and outpatient visits to TIBDN hospitals, time of *bla*_VIM-1_ detection, healthcare visits abroad, and MLST clusters are shown in Fig. 2. Of note, patients #3 and #4 from cluster I were hospitalized on the same ward in hospital C, but not in the same room or at the same time. Patients in cluster II were all linked to acute care hospital F, with patient #6 being the first identified patient; this outbreak has been described elsewhere^17^. The only potential direct link between the two clusters was that patient #6 (cluster II) underwent gastroscopy about 1 month after patient #2 (cluster I) had a sigmoidoscopy in the endoscopy unit at hospital B. Three out of four patients from cluster I lived in relative proximity to each other and to hospital C (Supplementary Fig. 1), whereas the site where the related surface water sample was taken was more distant. The index patient from cluster II, the related sewage water isolate and hospital F were all centrally located (Supplementary Fig. 1).

Chart reviews were performed on all 14 TIBDN patients with detection of *bla*_VIM-1_. Patients with (n = 7) and without (n = 7) known potential exposure were similar regarding risk factors, except for older age (87 *vs.* 71 years, P = 0.03) in those without explained acquisition. All three patients residing in long term care facilities (LTCFs) were in the group without explained acquisition (43% *vs.* 0%, P = 0.19), but all lived in different facilities (Supplementary Table 1, Supplementary Fig. 1). Six patients (43%) had an infection due to *bla*_VIM_-positive *E. hormaechei* (5 urinary tract infections; 1 blood stream infection).

### Susceptibility profiles and antimicrobial resistance genes

Susceptibility profiles and resistance mechanisms for clinical and environmental *bla*_VIM_-positive isolates are shown in Supplementary Tables 2 and 3. All isolates were resistant to at least one carbapenem. Ertapenem MICs ranged from 0.064 to ≥ 8 µg/ml (MIC_50_ and MIC_90_ of 2 and ≥ 8 µg/ml). For both meropenem and imipenem MICs ranged from 0.5 to ≥ 32 µg/ml (MIC_50_ of 4 and 8 µg/ml, respectively; MIC_90_ of 16 µg/ml for both). Only one isolate that was susceptible to meropenem (*C. freundii* Cfr-12) was also susceptible to cefepime: otherwise all isolates were resistant to all cephalosporins. All isolates but one were susceptible (or showed intermediate resistance, MIC = 8 µg/ml) to aztreonam (range 0.03 to 8 µg/ml; MIC_50_ and MIC_90_ of 2 and 4 µg/ml). Only one isolate, an extensively-drug resistant *K. pneumoniae* (Kpn-14), was highly resistant to aztreonam (MIC ≥ 256 µg/ml) and only susceptible to tigecycline and colistin.

**Table 2:** Characteristics of VIM plasmids.

Plasmid replicon	Plasmid size	Pairwise identity	Isolates	Description of isolates/patients	Relatedness to plasmid sequences in GenBank
IncR	~ 33 kb	99.2%	Eho-6 to Eho-11, Eho-E2	All 7 isolates in cluster II (hospital outbreak with 6 patients plus 1 surface water isolate)	Nucleotide identity in relatively short length of DNA encoding for replication, maintenance and stability of the plasmid (Suppl. Figures 2 to 6)
	~ 38 kb	99.5%	Eho-2, Eho-4, Eho-5	3 patient isolates, cluster I	
	~ 65 kb, ~ 83 kb, ~ 96 kb	88.1%	Eho-E1, Eho-E3, Eho-E4	3 water isolates, cluster I	
IncHI2	~ 325 kb	–	Eho-2	1 patient isolate, cluster I	99.9% nucleotide identity (90% of query cover) with plasmids carrying *bla*_VIM-1_ in *Salmonella enterica* serovar Infantis in Germany (pRH-R27, acc. number LN555650, ~ 299 kb; and pSE15-SA01028, CP026661, ~ 311 kb) (Suppl. Figure 7)
IncFII/IncFIIB	~ 178 kb	–	Eho-1	Patient #1, previously hospitalized in Split, Croatia	100% nucleotide identity (84% of query cover) with a 210 kb *bla*_KPC_-carrying plasmid found in *E. hormaechei* (CP12169, from New York City, USA)
IncN/IncR	~ 81 kb	97.8%	Cfr-13a, Kpn-13b	2 isolates from patient #13 (no identified risk)	Almost 100% nucleotide identity (64% of query cover) with plasmids carrying *bla*_NDM_ (CP040178, from China) and *bla*_IMP_ (KU726588, KT982615, KT982616, KT989376, from Hong Kong)
IncA/C2	~ 152 kb	–	Kpn-14	Patient #14, previously hospitalized in Egypt	99.98% nucleotide identity, 92% of query cover, with pKPC_CAV1344, CP011622, ~ 176 kb
IncA/C	~ 153 kb	–	Eco-15	Patient #15 (no identified risk)	99.98% nucleotide identity, 98% of query cover, with VIM plasmids found in *Aeromonas* sp., MH220284, ~ 165 kb, and *K. pneumoniae*, KY882285, ~ 156 kb
None identified	~ 30 kb	–	Cfr-12	Patient #12, previously hospitalized in Aveiro, Portugal	99.99% identity with pJB12 (KX889311), detected in 4 *Pseudomonas aeruginosa* from hospitalized patients in Portugal (see text) (28, 29)

In addition to *bla*_VIM_ genes, all *E. hormaechei* in cluster I (except Eho-2) as well as the environmental Eho-E3 and Eho-E4 also had a plasmid-mediated AmpC (*bla*_ACC-1_); similarly, *bla*_CTX-M-15_ was found in all *E. hormaechei* in cluster II. All these isolates harbouring *bla*_ACC-1_ and *bla*_CTX-M-15_ displayed reduced susceptibility to aztreonam (MIC 1.5 to 4 µg/ml). Isolate Eho-1 had only an additional *bla*_OXA-1_, encoding for a narrow spectrum ß-lactamase, but also displayed an aztreonam MIC of 8 µg/ml. Two or more aminoglycoside-modifying enzyme genes were described in all the isolates, but only five isolates were resistant to gentamicin, and only three (from the three patients with a history of hospitalization in other countries) were resistant to amikacin. All isolates carried at least one plasmid-mediated quinolone resistance mechanism, but only nine isolates, which were also *gyrA*/*parC* double mutants, were highly resistant to nalidixic acid (MIC ≥ 256 µg/ml) and ciprofloxacin (≥ 32 µg/ml). All isolates were susceptible to tigecycline and colistin, and only one isolate (Kpn-14) was resistant to fosfomycin despite the fact that all cluster I isolates were *fosA* positive (see Supplementary Table 3).

### Characterisation of plasmids carrying *bla*_VIM_ genes

**Figure 3:**
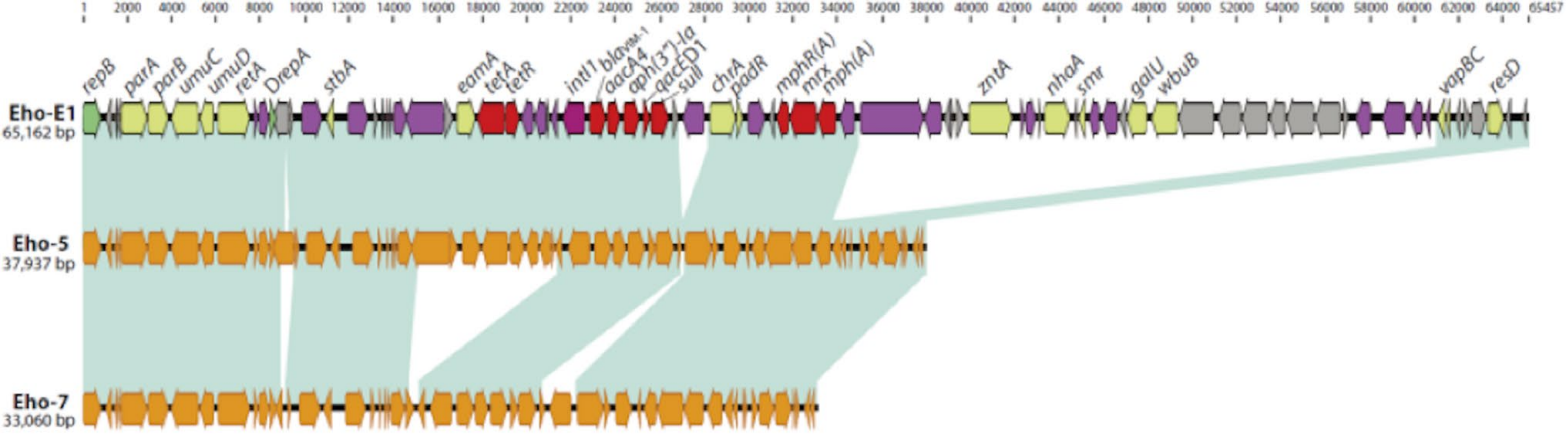
Comparison of IncR plasmids between *bla*_VIM-1_ carrying *E. hormaechei* isolates from cluster I (environmental isolate Eho-E1 and clinical isolate Eho-5) and cluster II (clinical isolate Eho-7); for more information about IncR plasmids, see Supplementary Figs. 2–6.

Features of VIM plasmids are detailed in Table 2 and Supplementary Figs. 2–7. Of fifteen VIM plasmids among *E. hormaechei* isolates, thirteen belonged to the IncR replicon type. Cluster I included one IncHI2 and four IncR plasmids, three of which had similar size (~ 38 kb) and shared high nucleotide identity (99.5%) (Supplementary Fig. 2). The remaining IncR plasmid in cluster I (environmental isolate Eho-E1 recovered from surface water in 2015) was larger (~ 65 kb) (Fig. 3 and Supplementary Figs. 5 and 6). The plasmids in cluster II (including one environmental isolate Eho-E2) were homogeneous in size (~ 33 kb) and shared high nucleotide pairwise identity (99.2%) (Supplementary Fig. 3). Comparison of IncR plasmids from cluster I and cluster II identified a ~ 6.4 kb DNA fragment (encoding for the tetracycline efflux protein TetA and its repressor TetR) in cluster I but not in the cluster II plasmid (Supplementary Fig. 4). The two remaining environmental *E. hormaechei* isolates, recovered from the same surface water site on the same day, were also ST93 and carried IncR VIM plasmids of different sizes (Eho-E3, ~ 96 kb; Eho-E4, ~ 83 kb; 88.1% pairwise identity) (Supplementary Fig. 6), for which no significant alignments against the GenBank database were obtained.

One of the VIM-plasmids found in one clinical *E. hormaechei* isolate from cluster I (Eho-3, ST93) belonged to the IncHI2 (~ 325 kb) (Supplementary Fig. 7). Clinical isolate Eho-1 did not belong to any of the two previous clusters (it was typed as ST92) and harbored an IncFII/IncFIIB, *bla*_VIM-1_–plasmid of ~ 178 kb. Interestingly, this patient (patient 1) was found to be colonized upon transfer from Croatia, suggesting acquisition abroad. Indeed, the detection of the same sequence type in the same time period and institution has been documented^18,19^.

One *C. freundii* (Cfr-12, ST129) carried the *bla*_VIM-2_ gene in a 30 kb plasmid. It had almost 100% identity with pJB12 (KX889311), detected in four *P. aeruginosa* from inpatients of two different hospitals in Portugal^20,21^. pJB12 has the *bla*_VIM-2_-carrying In58 integron immersed in Tn*6352*, a transposon composed of the In58 and IS*Pa17* elements. Partial sequences of this In58 were found in GenBank, one from *C. freundii* (JX486753, uploaded in 2012) and the other from *P. aeruginosa* (KJ679406, uploaded in 2014), both from Aveiro, Portugal. Interestingly, the Ontario patient had a previous history of hospitalization in Aveiro in 2011, further supporting the hypothesis of acquisition abroad. However, since the Aveiro isolates and plasmids were not characterized, this possibility cannot be confirmed. It is also notable that even if the machinery for self-conjugation is incomplete, pJB12 might be mobilizable in the presence of a helper plasmid, a hypothesis that would be supported by the fact that this same plasmid was found in *P. aeruginosa* in Portugal and in *C. freundii* in Ontario, Canada (and potentially also in Portugal). The remaining *C. freundii* (Cfr-13) and *K. pneumoniae* (Kpn-13a) were colonizing the same patient (patient 13). The *bla*_VIM-1_ gene was identified in both cases on ~ 81 kb, IncN-IncR plasmids sharing 97.8% nucleotide identity, supporting the notion of intra-patient dissemination of this plasmid. The last two VIM-plasmids, with low nucleotide identity between them, were found in *K. pneumoniae* Kpn-14 and *E. coli* Eco-15 (Table 2).

All isolates carried the *bla*_VIM_ genes in In110 integrons (*bla*_VIM-1_-*aacA4′*-*aadA1b*), except one *C. freundii* (In58: *aacA7*-*bla*_VIM-2_-*aacC1*-*aacA4*) (Supplementary Table 2). A new integron (In1772) was identified in the environmental *E. hormaechei* Eho-E3 and Eho-E4. In1772 has a truncated *intI1* integrase gene, displaying a novel array of gene cassettes, similar to In110 (*bla*_VIM-1_-*aacA4*-10-*aadA1b*).

## Discussion

In Southern Ontario, Canada, autochthonous cases of VIM-1-producing *Enterobacteriaceae* have repeatedly been detected over a period of almost seven years. In this study, two clusters (thereof one hospital outbreak) with two different *E. hormaechei* sequence types but closely related plasmids suggest clonal spread and potential horizontal gene transfer as responsible for *bla*_VIM_ dissemination. The additional detection of identical *E. hormaechei* isolates in sewage and surface water suggests that the reservoirs may not solely be the gastrointestinal tract of patients. We could not identify common exposures among patients with unexplained VIM-acquisition. With the exception of a probable intra-patient horizontal dissemination of *bla*_VIM_ (patient 13), VIM-plasmids in clinical isolates from other bacterial species were not similar suggesting independent acquisition.

Our results suggest a common ancestral IncR plasmid for patient clusters I and II. Although it has been postulated that IncR plasmids are mobilizable because of their broad host range^22^, they may in fact be non-transferable and non-mobilizable due to the lack of a transfer system and a relaxase^23–25^. These features suggest that the evolution of these IncR plasmids occurred independently in clinical and environmental isolates belonging to the same clonal groups, with more structural stability among clinical isolates.

*E. hormaechei*, a member of the *E. cloacae* complex, was the most frequently isolated pathogen among *bla*_VIM_-positive *Enterobacteriaceae* and the only *Enterobacter* spp. carrying *bla*_VIM_ in our analysis. This association has also been found in an analysis of a collection of 170 *Enterobacter* isolates from around the globe, where *bla*_VIM_ was the most commonly detected carbapenemase^14^. *Enterobacter* spp. are colonizers of the human gastrointestinal tract, which is considered to be the primary reservoir for nosocomial infections. Such infections occur primarily in highly debilitated patients after prolonged hospitalization and exposure to antibiotics^26^. Numerous hospital outbreaks such as the one described in cluster II have been described^12,17,18,27^. Many reports from the USA and Europe have also identified LTCFs as reservoirs for CPE, including VIM^28–30^*.* Although three of four patients from cluster I were residents of LTCFs, they lived in three different facilities with different ownership separated by 5–50 km. In our setting, direct transfers between LTCFs are rare and are unlikely to provide a connection between these cases. However, indirect transmission via stays in acute care hospitals serving these institutions (such as hospitals B or C), or shared staff and equipment might explain acquisition of these strains. The similarity in characteristics of cases with explained and unexplained sources suggests that undetected colonized patients are the most likely sources of new acquisitions in our setting.

Common exposure to non-healthcare sources (to a food or environmental source) is another possible explanation for our findings. Interestingly, VIM is among the most frequently reported carbapenemases responsible for contamination of the hospital water environment, which may also be a source for new acquisitions^31^. Several studies have identified *bla*_VIM_ from non-healthcare sources such as sewage and surface waters^6–10^, drinking water^32^, sea gulls^33^, and from seafood^34,35^. Of note, reports from Europe have demonstrated the isolation of identical CPE from environmental water and patient samples^36, 37^, most likely acquired during recreational swimming^37^. We cannot determine from our study whether our water isolates are a result of contamination from colonized patients, or whether these water sources represent an environmental reservoir from which patients may acquire VIM. Geographical mapping of patient residences and environmental sampling did not reveal any clear association.

In addition to autochthonous cases, importation from hospitals with endemic or epidemic VIM-producing *Enterobacteriaceae* is also occurring. One third of patients with VIM-producing organisms had a hospital admission abroad in the year prior to detection; most of whom were repatriated from high-risk countries. The two cases with presumable VIM-acquisition in Croatia and Portugal, supported by high-resolving molecular data, highlight the importance of CPE screening and pre-emptive isolation in patients with a history of hospitalization in institutions with endemic or epidemic CPE.

A strength of our study is the availability of population-based CPE surveillance data. Together with the thorough patient workup this enabled us to get a complete picture of the local epidemiology and to reliably identify common exposures between patients. Furthermore, detailed microbiologic investigations including WGS allowed us to complement the epidemiological data.

Our study has limitations. The definition of explained and unexplained VIM-acquisition is arbitrary. We nevertheless think that considering both epidemiologic and microbiologic criteria for this definition assists in understanding the source and transmission of carbapenemases. Another limitation is the lack of patient information regarding non-healthcare exposures such as food or environment (e.g. exposure to environmental water), although the patients’ age and comorbidities make this scenario rather unlikely. Also, the number of environmental samples in our study was rather small.

In conclusion, VIM-1 producing *Enterobacteriaceae* are sporadically being reported in Ontario and mainly involve *E. hormaechei*. Dissemination of these pathogens is most likely due to undetected colonization and transmission in acute care and, potentially, LTCF. Further studies are needed to examine the role of LTCF and of environmental contamination in the local epidemiology of VIM-1.

## Methods

### Setting and sample sources

TIBDN has performed population-based surveillance of CPE in metropolitan Toronto and Peel Region from the time of their first identification in 2007^16^. Population-based surveillance was actively started in July 2014. To identify cases before July 2014, various data sources were consulted, including data from a voluntary surveillance program, microbiology laboratories, infection control departments, and Public Health Ontario Laboratories. For this analysis, we included all *bla*_VIM_-positive *Enterobacteriaceae* identified by TIBDN laboratories between October 2007 and January 2016. Four environmental VIM-positive *Enterobacter cloacae* complex isolates which were recovered in July–August 2015 from sewage and surface water in Toronto were also included^38^.

### Patient data

For patients colonized or infected with *bla*_VIM_-positive *Enterobacteriaceae*, travel history including healthcare abroad was collected by chart review and patient interview. Chart review was performed for all hospital admissions and outpatient visits in TIBDN hospitals from one year before first CPE detection until April 2016.

Data on epidemiological (e.g. LTCFs, previous hospital admissions and healthcare visits) and clinical risk factors (e.g. comorbidities, antibiotic exposure, medical interventions)—mainly based on reports from the literature—within 365 days before first VIM detection were collected^28,31,39^.

### Microbiology investigations

For identification of CPE, TIBDN laboratories adhere to a standardized diagnostic process^40^. Carbapenem-resistant enterobacterial isolates with phenotypic production of a carbapenemase underwent PCR screening for the most common carbapenemase genes (*bla*_VIM_, *bla*_NDM_, *bla*_KPC_, *bla*_OXA-48_, *bla*_IMP_, *bla*_GES_, *bla*_SME_, and *bla*_NMC_). Environmental sampling was performed as previously described^38^.

MALDI-TOF MS (bioMérieux VITEK-MS) was performed on all pure cultures to identify organisms to the species level. Antimicrobial susceptibility testing by Etest (bioMérieux, Marcy l’Etoile, France) or broth microdilution was performed and interpreted using the Clinical and Laboratory Standards Institute (CLSI) guidelines^41^, except in the case of colistin^42^. Because there are no breakpoints for tigecycline in the 2019 CLSI and EUCAST (only for *Escherichia coli* and *Citrobacter koseri*) guidelines, we used the 2018 EUCAST breakpoints (S ≤ 1 µg/ml; R > 2 µg/ml) for interpretation of species other than *E. coli*.

### Whole-genome sequencing and bioinformatics

Genomic DNA extraction, Illumina sequencing libraries and sequencing runs were performed as described^43^. Nanopore sequencing was performed on Oxford Nanopore Technologies (ONT) MinION device with chemistry 8 and flow cells FLO-MIN106 version R9.4. DNA was extracted using the MasterPure Complete DNA & RNA Purification kit (Epicenter Illumina, Wisconsin USA) with elution carried out to a final volume of 40 µL in TE buffer. Libraries for 12 isolates were prepared with the Rapid Barcoding Kit SQK-RBK004 starting with 400 ng of high molecular weight DNA from each isolate and according to Oxford Nanopore protocol (RBK_9054_V2_revE_23jan2018). Libraries were loaded and run for 48 h. Base calling was performed while sequencing or using Guppy. Nanoplot was used for quality control. Porechop (https://github.com/rrwick/Porechop) was used to split files by and to trim barcodes. Illumina-ONT hybrid assemblies were performed using Unicycler. Circular plasmid sequences were obtained from the hybrid assemblies. The assembled sequences were annotated using the RAST server (https://rast.nmpdr.org/rast.cgi) and the sequences compared in a pairwise fashion using BRIG^44^.

Resistance genes were detected using the StarAMR pipeline (https://github.com/phac-nml/staramr), which scans genome contigs against the ResFinder, PlasmidFinder, and PointFinder databases (Center for Genomic Epidemiology). Multilocus sequence typing (MLST) for *E. cloacae*, *E. coli* and *K. pneumoniae* were carried out in silico using assembled contigs (https://cge.cbs.dtu.dk/services/MLST/)^45^. Single-nucleotide polymorphism (SNP) analysis was conducted using a custom pipeline. Briefly, reads for all isolates were mapped against reference strain using SMALT software (v 0.7.6). The reference strains used are the follow: *Enterobacter cloacae* AR_0053 (GenBank BioSample: SAMN04014894; BioProject PRJNA292904); *Klebsiella pneumoniae* HS11286 (ASM24018v2, GenBank BioSample: SAMN02602959, BioProject: PRJNA78789); *Citrobacter freundii* CFNIH1 (ASM64851v1, GenBank BioSample: SAMN02713684, BioProject: PRJNA202883). SNP calling was performed using Freebayes with: min-base-quality 30, minmapping-quality 30, min-alternate-fraction 0.75, read-snp-limit 10, min-coverage 15. Additional variant confirmation was done using the SAMtools mpileup tool. Repetitive regions were removed by using MUMmer. The meta-alignment of core informative positions was used to create a maximum likelihood tree using MEGA 7. Integron numbers were assigned by INTEGRALL, available at https://integrall.bio.ua.pt/^46^. The genome sequences for the isolates included in this study are deposited with NCBI, BioProject ID: PRJNA599404.

### Case–control study

A case–control study was performed among patients with *bla*_VIM-1_-positive *Enterobacteriaceae* to identify possible epidemiological risk factors for acquisition of *bla*_VIM-1_. Patients without a plausible source of acquisition were defined as cases, those with known risk as controls. Plausible sources of acquisition were defined as exposure as a patient in any hospital abroad OR spatiotemporal linkage (i.e. same hospital room within 3 months) to another patient in a TIBDN hospital with a VIM-containing isolate of the same species and MLST-type.

### Statistical analyses

We used SAS (SAS Institute, Cary, NC) for all statistical analyses. A two-tailed P-value < 0.05 was considered statistically significant. Categorical variables were reported as frequencies and proportions, continuous variables as median with range. For the case–control study, univariate analysis was performed using chi-square test or Fisher-exact, as appropriate, for dichotomous variables, and the Mann–Whitney *U* test for continuous variables.

### Ethics

Informed consent was obtained for patients with any specimen yielding CPE after January 2013, but consent was waived for those with positive specimens prior to 2013 only. This approach was approved by institutional review boards of all participating TIBDN hospitals and Public Health Ontario. The study was performed according to the World Medical Association Declaration of Helsinki.

## Supplementary information

Supplementary Information.
