# Supplementary material for: Dissemination of Verona Integron-encoded Metallo-β-lactamase among clinical and environmental Enterobacteriaceae isolates in Ontario, Canada
Source: Sci Rep. 2020 Oct 29;10:18580. doi: 10.1038/s41598-020-75247-7 (PMC7596063; doi:10.1038/s41598-020-75247-7)
Supplement: Supplementary file 1 — Supplementary Information. [file 41598_2020_75247_MOESM1_ESM.pdf]

# Dissemination of Verona Integron–encoded Metallo-β-Lactamase among Clinical and Environmental Enterobacteriaceae Isolates in Ontario, Canada

Philipp Kohler, Nathalie Tijet, Hyunjin C. Kim, Jennie Johnstone, Tom Edge, Samir N. Patel, Christine Seah, Barbara Willey, Brenda Coleman, Karen Green, Irene Armstrong, Kevin Katz, Matthew P. Muller, Jeff Powis, Susan M. Poutanen, David Richardson, Alicia Sarabia, Andrew Simor, Allison McGeer, Roberto G. Melano, for the Toronto Invasive Bacterial Diseases Network (TIBDN)

## Supplementary Figures and Tables

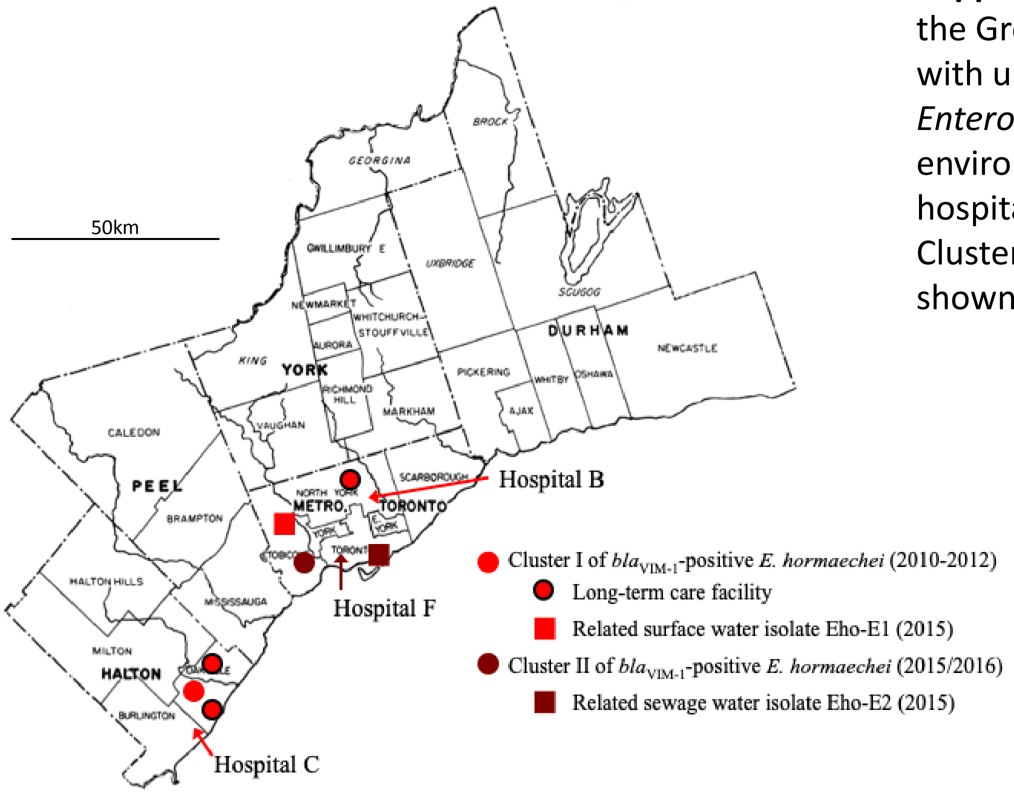


**Supplementary Figure 1.** Place of residence within the Greater Toronto Area, Canada, of five patients with unexplained acquisition of $bla_{VIM-1}$-positive *Enterobacter hormaechei* (circles) and site of environmental water sampling (squares). Involved hospitals (arrows) are depicted by arrows. Clustering based on multilocus sequence typing is shown in bright (cluster I) and dark red (cluster II).

| | Isolate | Type | Species | Enzyme | Source | MLST | Plasmid | | Integron |
|---|---|---|---|---|---|---|---|---|---|
| | | | | | | | Size (bp) | Replicon | |
| **CLUSTER I** | Eho-2 | Clinical | *E. hormaechei* | VIM-1 | Urine | 93 | 37,703 | IncR | In110 |
| | Eho-3 | Clinical | *E. hormaechei* | VIM-1 | Urine | 93 | 325,397 | IncHI2 | In110 |
| | Eho-4 | Clinical | *E. hormaechei* | VIM-1 | Urine | 93 | 37,460 | IncR | In110 |
| | Eho-5 | Clinical | *E. hormaechei* | VIM-1 | Urine | 93 | 37,715 | IncR | In110 |
| | Eho-E1 | **Environ** | *E. hormaechei* | VIM-1 | Surface water | 93 | 65,162 | IncR | In110 |

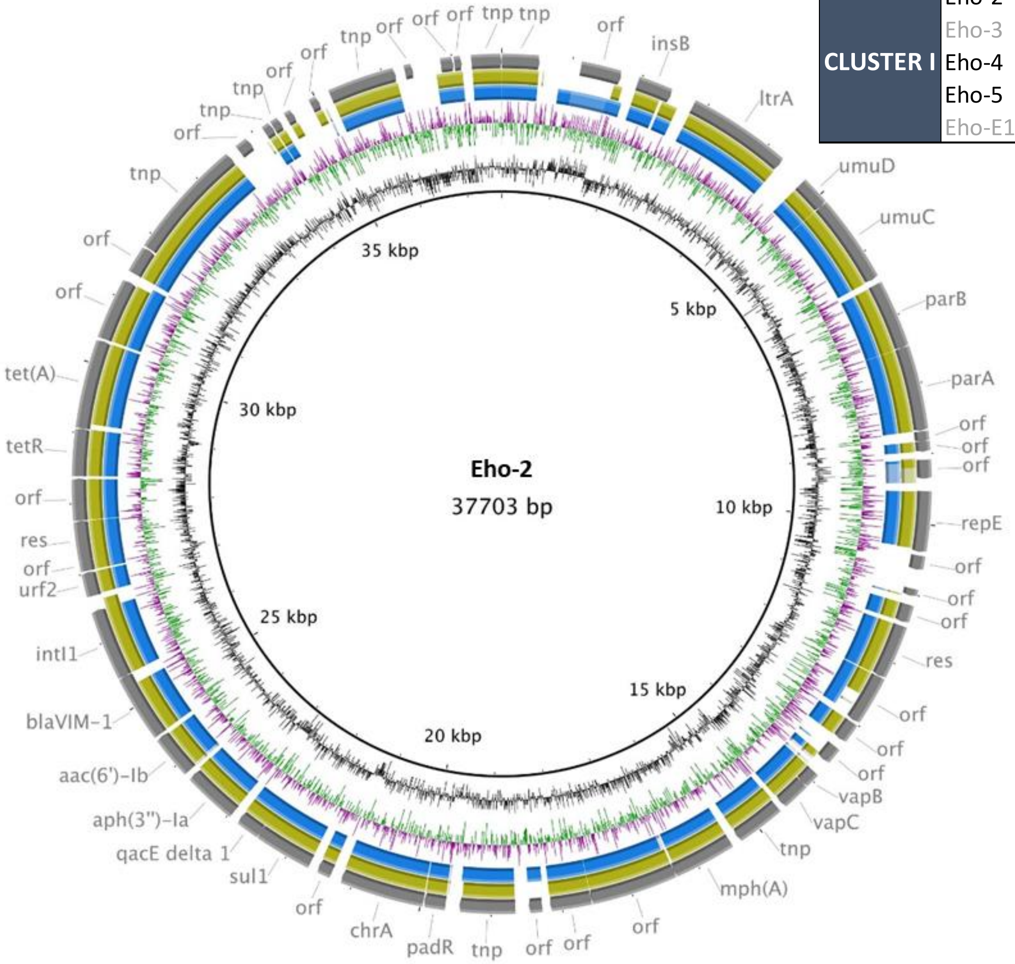


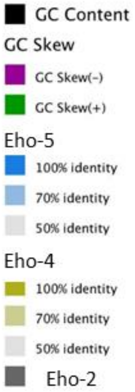


| | Eho-5 | Eho-4 | Eho-2 |
|---|---|---|---|
| **Eho-5** | | 99.89% | 98.90% |
| **Eho-4** | | | 99.01% |
| **Eho-2** | | | |

**Suppl. Figure 2.** ~38 Kb plasmids belonging to Cluster I. Pairwise identity: 99.5%

| | Isolate | Type | Species | Enzyme | Source | MLST | Plasmid Size (bp) | Plasmid Replicon | Integron |
|---|---|---|---|---|---|---|---|---|---|
| CLUSTER II | Eho-6 | Clinical | *E. hormaechei* | VIM-1 | Rectal | 269 | 33,171 | IncR | In110 |
| | Eho-7 | Clinical | *E. hormaechei* | VIM-1 | Urine | 269 | 33,060 | IncR | In110 |
| | Eho-8 | Clinical | *E. hormaechei* | VIM-1 | Dialysis fluid | 269 | 32,990 | IncR | In110 |
| | Eho-9 | Clinical | *E. hormaechei* | VIM-1 | Dialysis fluid | 269 | 33,024 | IncR | In110 |
| | Eho-10 | Clinical | *E. hormaechei* | VIM-1 | Rectal | 269 | 33,171 | IncR | In110 |
| | Eho-11 | Clinical | *E. hormaechei* | VIM-1 | Rectal | 269 | 32,697 | IncR | In110 |
| | Eho-E2 | Environ | *E. hormaechei* | VIM-1 | Sewage water | 269 | 33,171 | IncR | In110 |

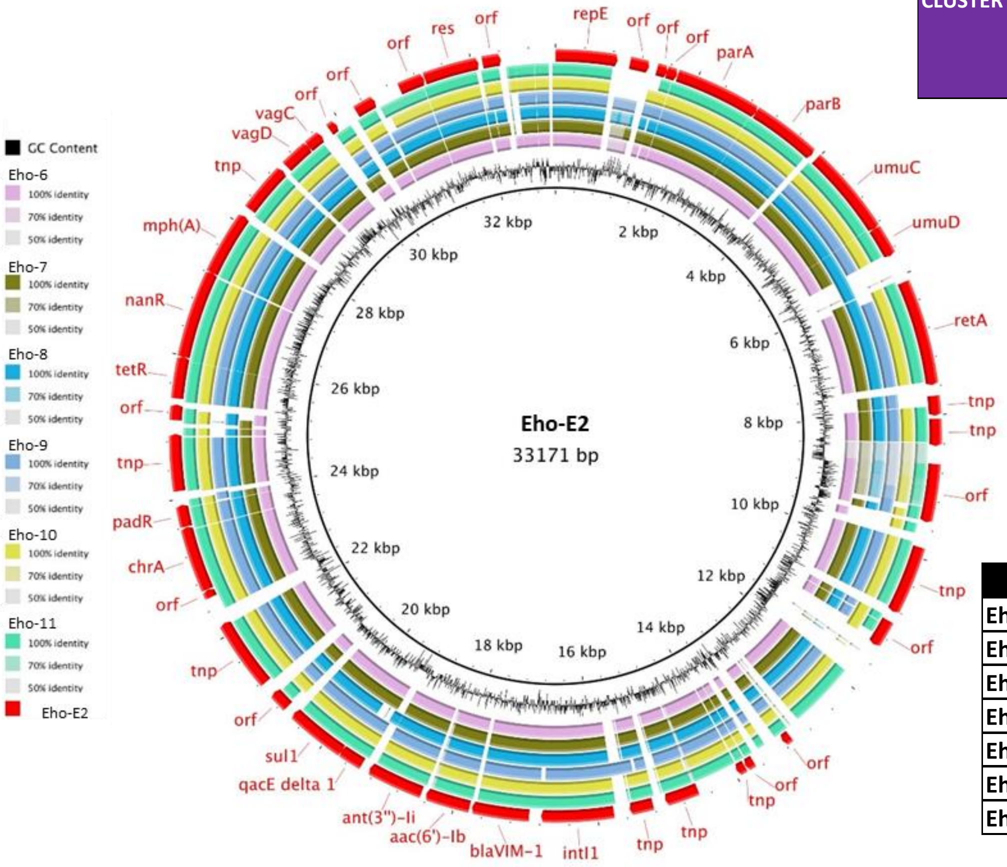


**Suppl. Figure 3.** ~33 Kb plasmids belonging to Cluster II. Pairwise identity: 99.2%

| | Eho-7 | Eho-9 | Eho-11 | Eho-10 | Eho-E2 | Eho-6 | Eho-8 |
|---|---|---|---|---|---|---|---|
| **Eho-7** | | 99.89% | 98.90% | 99.23% | 99.67% | 99.33% | 99.33% |
| **Eho-9** | | | 99.01% | 99.34% | 99.55% | 99.22% | 99.44% |
| **Eho-11** | | | | 99.66% | 98.57% | 98.24% | 99.09% |
| **Eho-10** | | | | | 98.90% | 98.57% | 99.43% |
| **Eho-E2** | | | | | | 99.67% | 99.42% |
| **Eho-6** | | | | | | | 99.09% |
| **Eho-8** | | | | | | | |

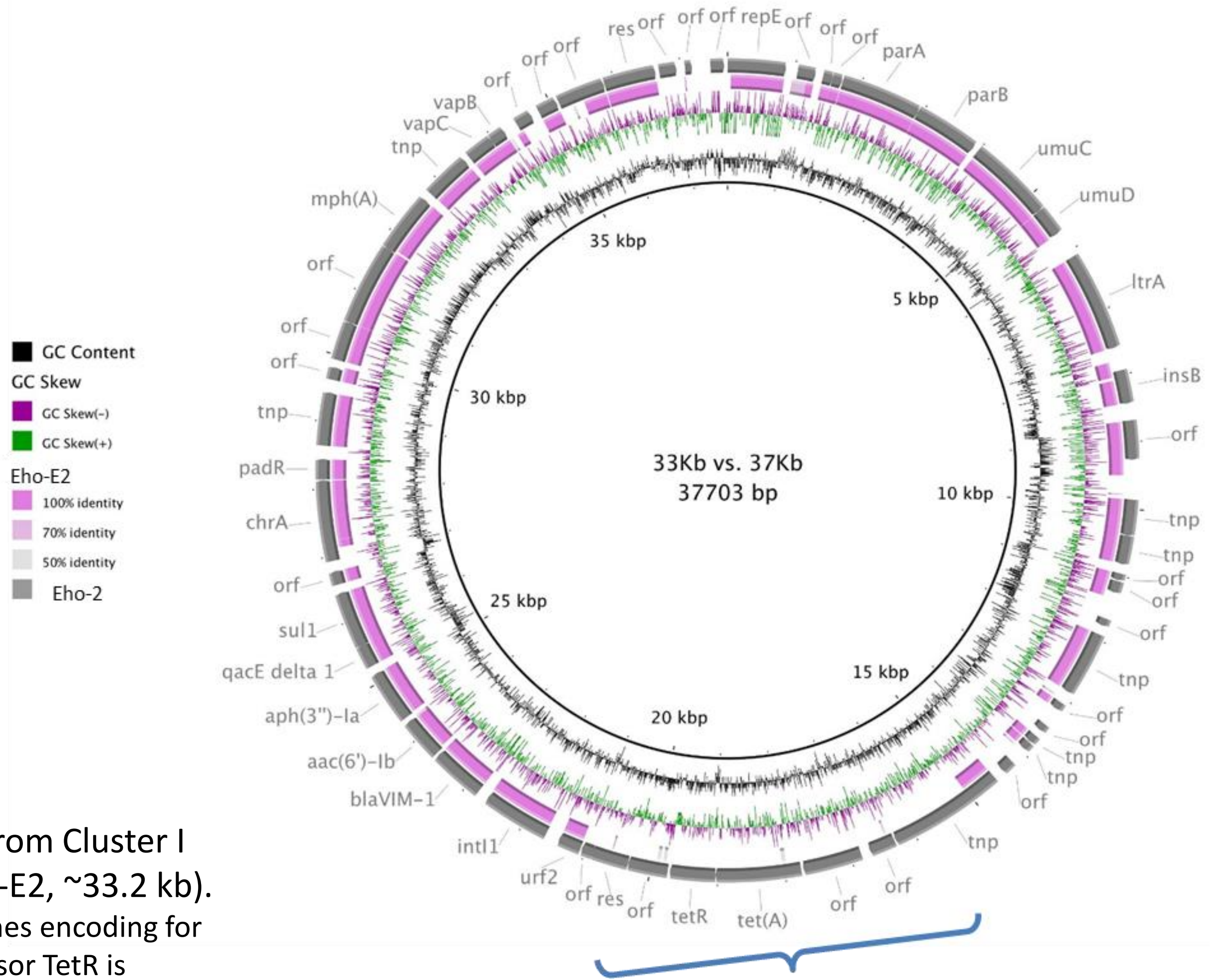


**Suppl. Figure 4.** Comparison of plasmids from Cluster I (Eho-2, ~37.7 kb) vs. Cluster II (isolate Eho-E2, ~33.2 kb).
A ~6.3 kb DNA fragment containing the *tetAR* genes encoding for the tetracycline efflux protein TetA and its repressor TetR is missing in Eho-E2.

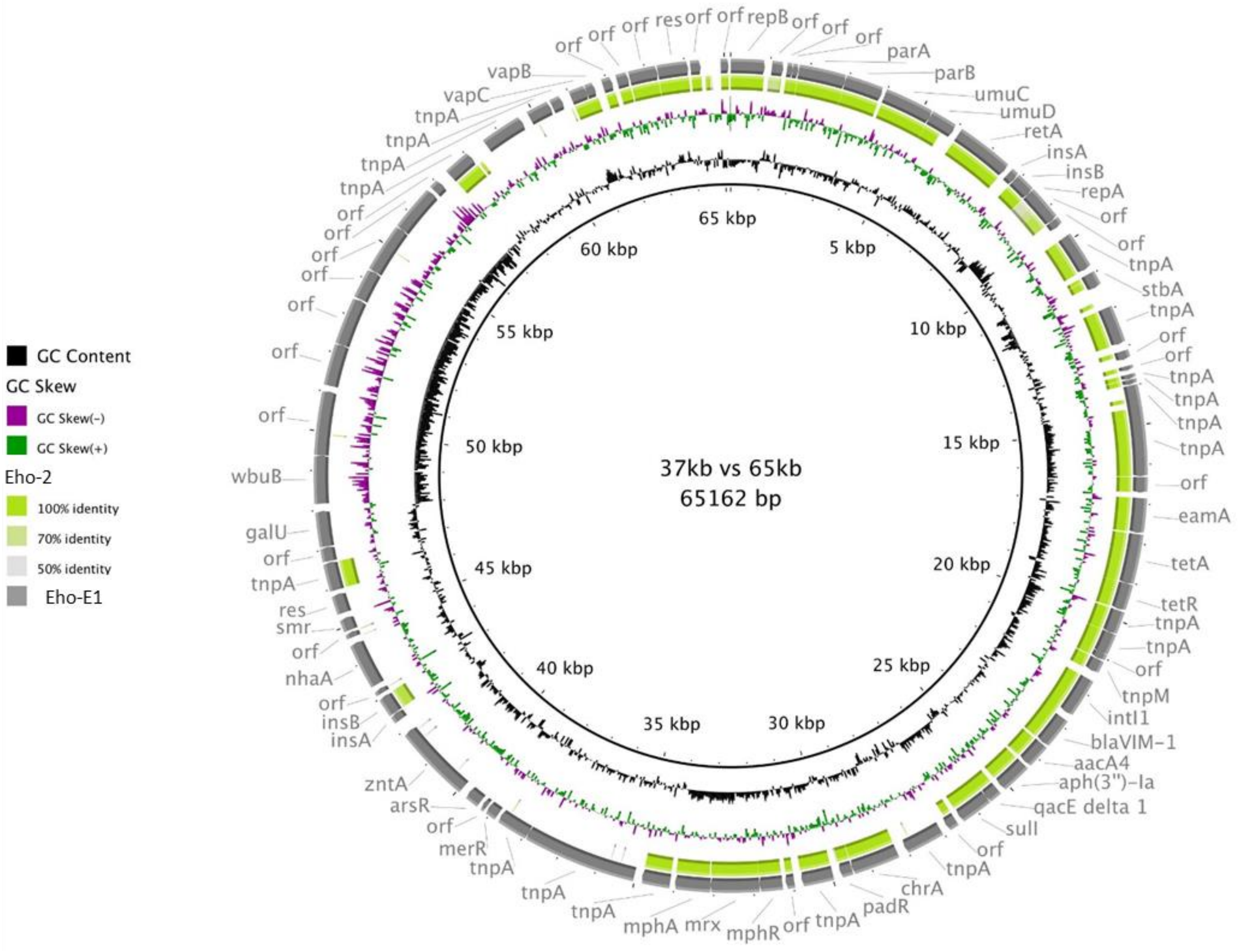


**Suppl. Figure 5.** Comparison of IncR plasmids from Cluster I (Eho-2, ~37.7 kb, vs. Eho-E1, ~65.2 kb)

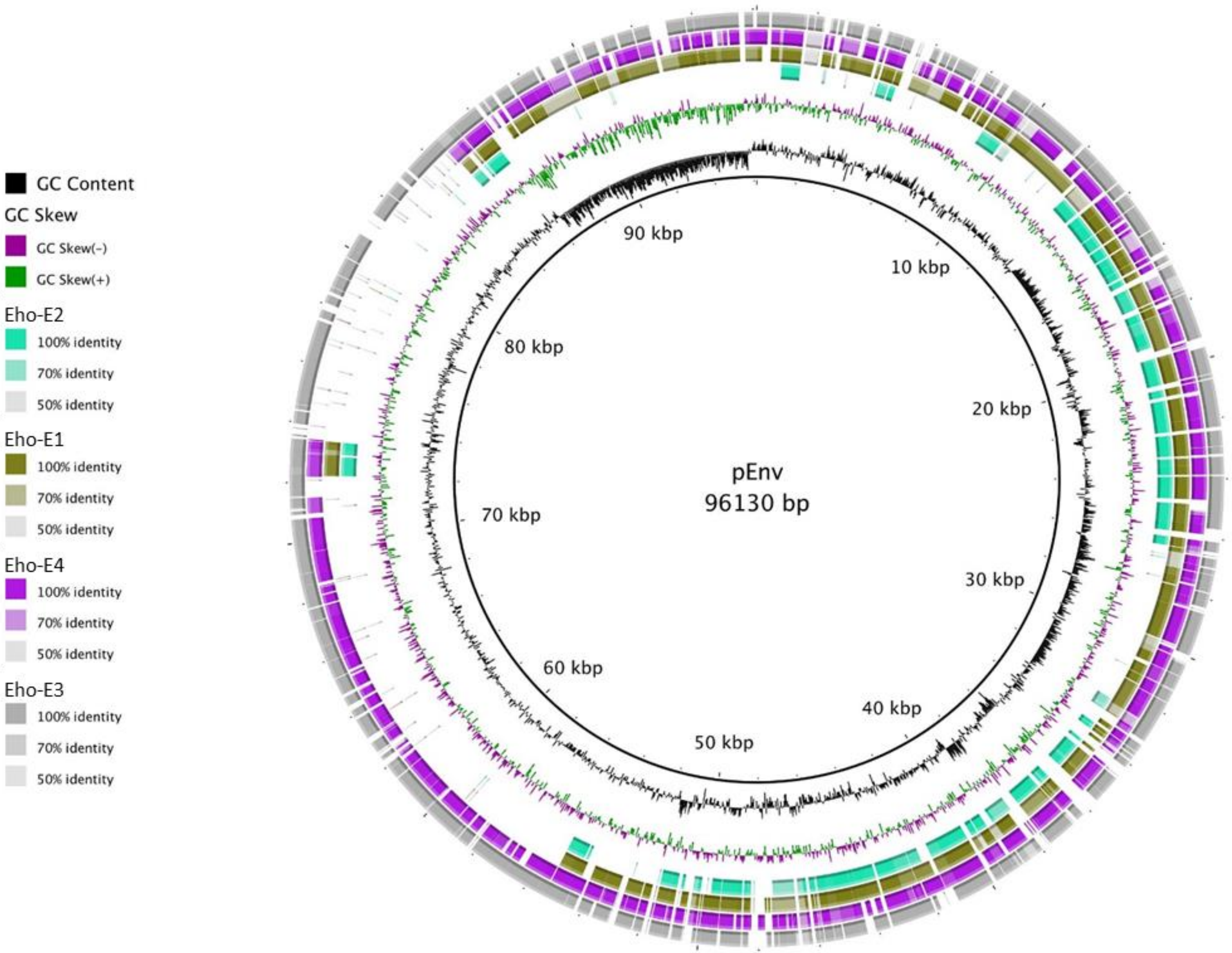


**Suppl. Figure 6.** *bla*$_{VIM-1}$, IncR plasmids from environmental isolates

**Suppl. Figure 7.** IncHI2 VIM-plasmid (~325 kb) found in clinical *E. hormaechei* isolate Eho-3 (from cluster I, ST93).

Comparison with pRH-R27 (GenBank acc. number LN555650, ~299 kb) and pSE15-SA01028 (CP026661, ~311 kb), *bla*$_{VIM-1}$-carrying plasmids found in *Salmonella enterica* serovar Infantis from Germany (99.92 %; 90% of query cover). A ~32 kb fragment (including a putative *lac* operon) disrupting an open reading frame encoding for a phosphoadenosine phosphosulfate reductase was detected in pEho-3. The lack of mobile elements flanking this fragment suggest a recombination event.

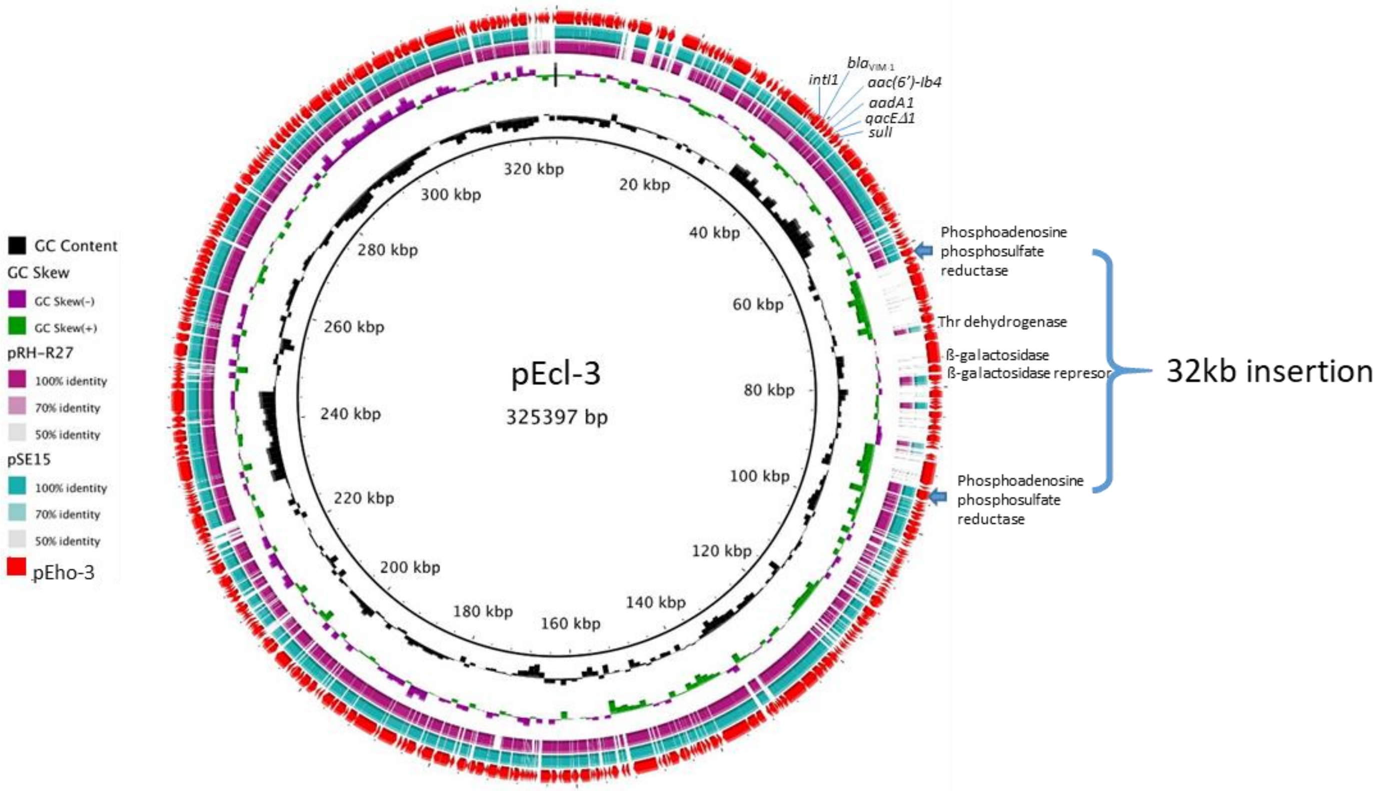

**Table S1. Risk profile of patients with explained (i.e. healthcare visit abroad or epidemiologically and microbiologically established link) and patients with unexplained acquisition of blaVIM-1-positive Enterobacteriaceae**

| | All patients (N=14) | Explained (N=7) | Unexplained (N=7) | P-value |
|---|---|---|---|---|
| **Demographics** | | | | |
| Male sex (%) | 7 (50.0) | 5 (71.4) | 2 (28.6) | 0.29 |
| Age in years, median (range) | 76.5 (24-90) | 71 (24-80) | 87 (65-90) | **0.03** |
| Clinical samples | 8 (57.1) | 4 (57.1) | 4 (57.1) | 1 |
| **Comorbidities** | | | | |
| Charlson score, median (range) | 3 (0-7) | 4 (0-5) | 2 (1-7) | 0.75 |
| Diabetes mellitus | 5 (35.7) | 4 (57.1) | 1 (14.3) | 0.27 |
| Cancer | 4 (28.6) | 1 (14.3) | 3 (42.9) | 0.56 |
| Chronic kidney disease | 5 (35.7) | 3 (42.9) | 2 (28.6) | 1 |
| Dialysis | 3 (21.4) | 3 (42.9) | 0 (0.0) | 0.19 |
| Dementia | 5 (35.7) | 1 (14.3) | 4 (57.1) | 0.27 |
| **Epidemiological exposures*** | | | | |
| Community-acquired† | 1 (7.1) | 0 (0.0) | 1 (14.3) | 0.56 |
| Nursing home resident | 3 (21.4) | 0 (0.0) | 3 (42.9) | 0.19 |
| Intensive care stay | 6 (42.9) | 4 (57.1) | 2 (28.6) | 0.59 |
| Outpatient visits, median (range) | 2 (0-8) | 1 (0-8) | 3 (0-8) | 0.9 |
| **Antibiotic resistant pathogens*** | | | | |
| MRSA | 2 (14.3) | 0 (0) | 2 (28.6) | 0.46 |
| ESBL | 4 (28.6) | 1 (14.3) | 3 (42.9) | 0.56 |
| **Medical interventions*** | | | | |
| Colonoscopy | 2 (14.3) | 1 (14.3) | 1 (14.3) | 1 |
| Gastroscopy | 3 (21.4) | 1 (14.3) | 2 (28.6) | 1 |
| Foley catheter | 7 (50.0) | 3 (42.9) | 4 (57.1) | 1 |
| Cystoscopy | 2 (14.3) | 1 (14.3) | 1 (14.3) | 1 |
| Surgery | 4 (28.6) | 0 (0.0) | 4 (57.1) | 0.07 |
| Central venous catheter | 3 (21.4) | 3 (42.9) | 0 (0.0) | 0.19 |
| Blood transfusion | 3 (21.4) | 1 (14.3) | 2 (28.6) | 1 |
| **Medication*** | | | | |
| Antibiotics | 5 (35.7) | 7 (100.0) | 5 (71.4) | 0.46 |
| Proton-Pump Inhibitors | 6 (42.9) | 3 (42.9) | 3 (42.9) | 1 |

*All within 1 year before VIM detection

†No previous hospital admission, no nursing home resident, no dialysis

**Table S2. Characteristics of the VIM-positive isolates included in this study**

| | Isolate | Type | Species | Enzyme | Source | Isolation date | MLST | Plasmid Size (bp) | Plasmid Replicon | Integron |
|---|---|---|---|---|---|---|---|---|---|---|
| | Eho-1 | Clinical | *E. hormaechei* | VIM-1 | Rectal | 1-Sep-2012 | 92 | 178'304 | IncFIB-IncFII | In110 |
| **CLUSTER I** | Eho-2 | Clinical | *E. hormaechei* | VIM-1 | Urine | 12-Jul-2011 | 93 | 37'703 | IncR | In110 |
| | Eho-3 | Clinical | *E. hormaechei* | VIM-1 | Urine | 10-Aug-2011 | 93 | 325'397 | IncHI2 | In110 |
| | Eho-4 | Clinical | *E. hormaechei* | VIM-1 | Urine | 18-Feb-2012 | 93 | 37'460 | IncR | In110 |
| | Eho-5 | Clinical | *E. hormaechei* | VIM-1 | Urine | 20-Jun-2012 | 93 | 37'715 | IncR | In110 |
| | Eho-E1 | Environ | *E. hormaechei* | VIM-1 | Surface water | 4-Aug-2015 | 93 | 65'162 | IncR | In110 |
| **CLUSTER II** | Eho-6 | Clinical | *E. hormaechei* | VIM-1 | Rectal | 25-Feb-2014 | 269 | 33'171 | IncR | In110 |
| | Eho-7 | Clinical | *E. hormaechei* | VIM-1 | Urine | 2-Jul-2014 | 269 | 33'060 | IncR | In110 |
| | Eho-8 | Clinical | *E. hormaechei* | VIM-1 | Dialysis fluid | 2-Jun-2015 | 269 | 32'990 | IncR | In110 |
| | Eho-9 | Clinical | *E. hormaechei* | VIM-1 | Dialysis fluid | 27-Sep-2015 | 269 | 33'024 | IncR | In110 |
| | Eho-10 | Clinical | *E. hormaechei* | VIM-1 | Rectal | 18-Jan-2016 | 269 | 33'171 | IncR | In110 |
| | Eho-11 | Clinical | *E. hormaechei* | VIM-1 | Rectal | 18-Jan-2016 | 269 | 32'697 | IncR | In110 |
| | Eho-E2 | Environ | *E. hormaechei* | VIM-1 | Sewage water | 23-Jul-2015 | 269 | 33'171 | IncR | In110 |
| **Other species** | Cfr-12 | Clinical | *C. freundii* | VIM-2 | Sputum | 28-Feb-2014 | 129 | 30'361 | No hit found | In58 |
| | Cfr-13a | Clinical | *C. freundii* | VIM-1 | Rectal | 21-Dec-2015 | New | 81'066 | IncN-IncR | In110 |
| | Kpn-13b | Clinical | *K. pneumoniae* | VIM-1 | Rectal | 14-Dec-2015 | 17 | 81'960 | IncN-IncR | In110 |
| | Kpn-14 | Clinical | *K. pneumoniae* | VIM-1 | Rectal | 29-Sep-2014 | 376 | 150'505 | IncA/C2 | In110 |
| | Eco-15 | Clinical | *E. coli* | VIM-1 | Rectal | 26-Nov-2015 | 131 | 152'546 | IncA/C | In110 |
| **Other Ecl env isolates** | Eho-E3 | Environ | *E. hormaechei* | VIM-1 | Surface water | 4-Aug-2015 | 93 | 96,194 bp | IncR | In1772 |
| | Eho-E4 | Environ | *E. hormaechei* | VIM-1 | Surface water | 4-Aug-2015 | 93 | 83,090 bp | IncR | In1772 |

*MIC, Minimal Inhibitory Concentration

FOX, Cefoxitin; CAZ, Ceftazidime; CTX, Cefotaxime; FEP, Cefepime; AZT, Aztreonam; ETP, Ertapenem; MEM, Meropenem; IMI, Imipenem; AMK, Amikacin; GEN, Gentamicin; NAL, Nalidixic acid; CIP, Ciprofloxacin; TGC, Tigecyclin; FOS, Fosfomycin; SXT, Sulfamethoxazol-Trimethoprim; COL, Colistin

**Table S2. Characteristics of the VIM-positive isolates included in this study**

| | Isolate | MIC* (µg/ml) | | | | | | | | | | | | | | | |
|---|---|---|---|---|---|---|---|---|---|---|---|---|---|---|---|---|---|
| | | FOX | CAZ | CTX | FEP | AZT | ETP | MEM | IMI | AMK | GEN | NAL | CIP | TGC | FOS | SXT | COL |
| | Eho-1 | ≥256 | ≥256 | ≥256 | 96 | 8 | 2 | 4 | 12 | 64 | 96 | 32 | 6 | 0.5 | 24 | ≥32 | 0.125 |
| CLUSTER I | Eho-2 | 256 | ≥256 | 192 | 32 | 0.12 | ≥8 | 16 | 8 | 2 | 24 | ≥256 | ≥32 | 1 | 32 | ≥32 | 0.125 |
| | Eho-3 | ≥256 | ≥256 | ≥256 | 256 | 4 | ≥8 | 8 | 16 | 3 | 2 | ≥256 | ≥32 | 0.25 | 6 | ≥32 | 0.125 |
| | Eho-4 | ≥256 | ≥256 | ≥256 | 256 | 1 | 8 | 16 | 8 | 2 | 32 | ≥256 | ≥32 | 0.75 | 32 | ≥32 | 0.094 |
| | Eho-5 | ≥256 | ≥256 | 256 | 64 | 4 | ≥8 | 12 | 16 | 3 | 96 | ≥256 | ≥32 | 0.5 | 12 | ≥32 | 0.125 |
| | Eho-E1 | ≥256 | ≥256 | ≥256 | 64 | 2 | ≥8 | 16 | 12 | 3 | 1 | ≥256 | ≥32 | 0.5 | 24 | ≥32 | 0.094 |
| CLUSTER II | Eho-6 | ≥256 | ≥256 | 128 | 24 | 2 | 4 | 1.5 | 6 | 8 | 2 | 16 | 2 | 0.19 | 0.38 | ≥32 | 0.094 |
| | Eho-7 | ≥256 | ≥256 | 256 | 24 | 4 | 2 | 8 | 8 | 8 | 2 | 16 | 3 | 0.19 | 0.094 | ≥32 | 0.125 |
| | Eho-8 | ≥256 | ≥256 | 128 | 24 | 2 | 2 | 12 | 8 | 6 | 2 | 8 | 3 | 0.125 | 0.25 | ≥32 | 0.094 |
| | Eho-9 | ≥256 | ≥256 | 256 | 64 | 2 | 2 | 2 | 4 | 6 | 2 | 12 | 4 | 0.19 | 0.5 | ≥32 | 0.094 |
| | Eho-10 | ≥256 | ≥256 | 128 | 48 | 2 | 2 | 1.5 | 4 | 2 | 1.5 | 12 | 0.75 | 0.25 | 8 | ≥32 | 0.094 |
| | Eho-11 | ≥256 | ≥256 | 192 | 32 | 2 | 2 | 1.5 | 4 | 12 | 3 | 16 | 4 | 0.38 | 0.75 | ≥32 | 0.094 |
| | Eho-E2 | ≥256 | ≥256 | 192 | 64 | 0.25 | 2 | 1.5 | 16 | 8 | 2 | 16 | 2 | 0.19 | 12 | ≥32 | 0.094 |
| Other species | Cfr-12 | ≥256 | 24 | 64 | 1 | 2 | 2 | 0.5 | 1.5 | 48 | ≥1024 | ≥256 | ≥32 | 0.38 | 0.5 | 0.5 | 0.125 |
| | Cfr-13a | ≥256 | ≥256 | 256 | 64 | 0.06 | 0.25 | 1 | 4 | 1.5 | 1.5 | 8 | 0.25 | 0.094 | 0.5 | ≥32 | 0.094 |
| | Kpn-13b | 128 | ≥256 | 48 | 12 | 0.03 | 1 | 1.5 | 8 | 2 | 1.5 | 8 | 1 | 0.19 | 32 | ≥32 | 0.125 |
| | Kpn-14 | ≥256 | ≥256 | ≥256 | ≥256 | ≥256 | ≥8 | ≥32 | ≥32 | 128 | 64 | ≥256 | ≥32 | 0.25 | ≥1024 | ≥32 | 0.125 |
| | Eco-15 | 32 | 48 | 8 | 4 | 8 | 0.06 | 16 | 0.5 | 2 | 1 | 6 | 0.032 | 0.094 | 1 | ≥32 | 0.094 |
| Other Ecl env isolates | Eho-E3 | ≥256 | ≥256 | ≥256 | 64 | 4 | ≥8 | 12 | 8 | 4 | 1.5 | ≥256 | ≥32 | 1 | 24 | ≥32 | 0.094 |
| | Eho-E4 | ≥256 | ≥256 | 256 | 64 | 2 | ≥8 | 4 | 8 | 4 | 1.5 | ≥256 | ≥32 | 0.75 | 16 | ≥32 | 0.094 |

*MIC, Minimal Inhibitory Concentration

FOX, Cefoxitin; CAZ, Ceftazidime; CTX, Cefotaxime; FEP, Cefepime; AZT, Aztreonam; ETP, Ertapenem; MEM, Meropenem; IMI, Imipenem; AMK, Amikacin; GEN, Gentamicin; NAL, Nalidixic acid; CIP, Ciprofloxacin; TGC, Tigecyclin; FOS, Fosfomycin; SXT, Sulfamethoxazol-Trimethoprim; COL, Colistin

**Table S3. Resistance genotypes and predicted phenotypes as well as plasmid replicon types of all isolates.**

| | Isolate ID | Genotype | Predicted Phenotype | Plasmid |
|---|---|---|---|---|
| | **Eho-1/GN1534** | aac(3)-IIa, aac(6')-Ib-cr, aac(6')-Ib3, aadA1, aph(3')-VIa, blaACT-16, blaOXA-1, blaVIM-1, catB4, dfrA14, qnrB1, sul1 | gentamicin, ciprofloxacin I/R, streptomycin, kanamycin, ampicillin, amoxicillin/clavulanic acid, cefoxitin, ceftriaxone, meropenem, chloramphenicol, trimethoprim, sulfisoxazole | IncFIB(pEhoA), IncFII(pEhoA), IncQ1 |
| CLUSTER I | **Eho-2/GN719** | aac(6')-Ib-cr, aac(6')-Ib3, aadA1, ant(2'')-Ia, aph(6)-Id, blaACT-7, blaTEM-1B, blaVIM-1, dfrA14, fosA, mph(A), qnrS1, strA, sul1, tet(A) | ciprofloxacin I/R, gentamicin, streptomycin, kanamycin, ampicillin, amoxicillin/clavulanic acid, cefoxitin, ceftriaxone, meropenem, trimethoprim, fosfomycin, erythromycin, azithromycin, sulfisoxazole, tetracycline | IncFII(Yp), IncN, IncR |
| | **Eho-3/GN738** | aac(6')-Ib-cr, aac(6')-Ib3, aadA1, aph(6)-Id, blaACC-1, blaACT-7, blaVIM-1, catA1, dfrA14, fosA, qnrS1, strA, sul1 | ciprofloxacin I/R, gentamicin, streptomycin, kanamycin, ampicillin, amoxicillin/clavulanic acid, cefoxitin, ceftriaxone, meropenem, chloramphenicol, trimethoprim, fosfomycin, sulfisoxazole | IncHI2, IncN |
| | **Eho-4/GN825** | aac(3)-IId, aac(6')-Ib-cr, aac(6')-Ib3, aadA1, aadA1b, ant(2'')-Ia, aph(6)-Id, blaACC-1, blaACT-7, blaTEM-1B, blaVIM-1, catA1, dfrA14, ere(A), fosA, mph(A), strA, sul1, tet(A) | gentamicin, ciprofloxacin I/R, streptomycin, kanamycin, ampicillin, amoxicillin/clavulanic acid, cefoxitin, ceftriaxone, meropenem, chloramphenicol, trimethoprim, erythromycin, azithromycin, fosfomycin, sulfisoxazole, tetracycline | Col156, IncFII(29), IncFII(Yp), IncHI2, IncHI2A, IncN, IncR |
| | **Eho-5/GN882** | aac(3)-IId, aac(6')-Ib-cr, aac(6')-Ib3, aadA1, aadA2, aph(6)-Id, blaACC-1, blaACT-7, blaTEM-1B, blaVIM-1, catA1, dfrA12, dfrA14, fosA, mph(A), qnrS1, strA, sul1, tet(A) | gentamicin, ciprofloxacin I/R, streptomycin, kanamycin, ampicillin, amoxicillin/clavulanic acid, cefoxitin, ceftriaxone, meropenem, chloramphenicol, trimethoprim, fosfomycin, erythromycin, azithromycin, sulfisoxazole, tetracycline | IncHI2, IncHI2A, IncN, IncR |
| | **Eho-E1/GN1533** | aac(6')-Ib-cr, aac(6')-Ib3, aadA1, aadA16, aph(6)-Id, ARR-3, blaACC-1, blaACT-7, blaVIM-1, dfrA27, floR, fosA, mph(A), qnrB6, strA, sul1, sul2, tet(A) | ciprofloxacin I/R, gentamicin, streptomycin, kanamycin, rifampicin, ampicillin, amoxicillin/clavulanic acid, cefoxitin, ceftriaxone, meropenem, trimethoprim, chloramphenicol, fosfomycin, erythromycin, azithromycin, sulfisoxazole, tetracycline | IncHI2, IncHI2A, IncN, IncR, IncX5 |

| | | | | |
|---|---|---|---|---|
| CLUSTER II | **Eho-6/GN1550** | aac(6')-Ib-cr, aac(6')-Ib3, aadA1, ant(3'')-Ia, aph(3'')-Ib, aph(6)-Id, blaCTX-M-15, blaOXA-1, blaTEM-1B, blaVIM-1, catA1, catB4, dfrA14, mph(A), qnrB1, qnrS1, sul1, sul2 | ciprofloxacin I/R, gentamicin, streptomycin, kanamycin, ampicillin, ceftriaxone, amoxicillin/clavulanic acid, cefoxitin, meropenem, chloramphenicol, chloramphenicol, trimethoprim, erythromycin, azithromycin, sulfisoxazole | IncHI2, IncHI2A, IncR |
| | **Eho-7/GN1157** | aac(6')-Ib-cr, aac(6')-Ib3, aadA1, ant(3'')-Ia, aph(3'')-Ib, aph(6)-Id, blaCTX-M-15, blaOXA-1, blaTEM-1B, blaVIM-1, catA1, catB4, dfrA14, mph(A), qnrB1, qnrS1, sul1, sul2 | ciprofloxacin I/R, gentamicin, streptomycin, kanamycin, ampicillin, ceftriaxone, amoxicillin/clavulanic acid, cefoxitin, meropenem, chloramphenicol, chloramphenicol, trimethoprim, erythromycin, azithromycin, sulfisoxazole | IncHI2, IncHI2A, IncR |
| | **Eho-8/GN1318** | aac(6')-Ib-cr, aac(6')-Ib3, aadA1, ant(3'')-Ia, aph(3'')-Ib, aph(6)-Id, blaCTX-M-15, blaOXA-1, blaTEM-1B, blaVIM-1, catA1, catB4, dfrA14, mph(A), qnrB1, qnrS1, sul1, sul2 | ciprofloxacin I/R, gentamicin, streptomycin, kanamycin, ampicillin, ceftriaxone, amoxicillin/clavulanic acid, cefoxitin, meropenem, chloramphenicol, chloramphenicol, trimethoprim, erythromycin, azithromycin, sulfisoxazole | IncHI2, IncHI2A, IncR |
| | **Eho-9/GN1494** | aac(6')-Ib-cr, aac(6')-Ib-cr, aac(6')-Ib-cr, aac(6')-Ib3, aadA1, ant(3'')-Ia, aph(3'')-Ib, aph(6)-Id, blaCTX-M-15, blaOXA-1, blaTEM-1B, blaVIM-1, catA1, catB4, dfrA14, mph(A), qnrB1, qnrS1, sul1, sul2 | gentamicin, ciprofloxacin I/R, streptomycin, kanamycin, ampicillin, ceftriaxone, amoxicillin/clavulanic acid, cefoxitin, meropenem, chloramphenicol, chloramphenicol, trimethoprim, erythromycin, azithromycin, sulfisoxazole | IncHI2, IncHI2A, IncR |
| | **Eho-10/GN1549** | aac(6')-Ib-cr, aac(6')-Ib3, aadA1, ant(3'')-Ia, aph(3'')-Ib, aph(6)-Id, blaCTX-M-15, blaTEM-1B, blaVIM-1, catA1, dfrA14, mph(A), qnrB1, qnrS1, sul1, sul2 | ciprofloxacin I/R, gentamicin, streptomycin, kanamycin, ampicillin, ceftriaxone, amoxicillin/clavulanic acid, cefoxitin, meropenem, chloramphenicol, trimethoprim, erythromycin, azithromycin, sulfisoxazole | IncHI2, IncHI2A, IncR |
| | **Eho-11/GN1555** | aac(6')-Ib-cr, aac(6')-Ib3, aadA1, ant(3'')-Ia, aph(3'')-Ib, aph(6)-Id, blaCTX-M-15, blaOXA-1, blaTEM-1B, blaVIM-1, catA1, catB4, dfrA14, mph(A), qnrB1, qnrS1, sul1, sul2 | ciprofloxacin I/R, gentamicin, streptomycin, kanamycin, ampicillin, ceftriaxone, amoxicillin/clavulanic acid, cefoxitin, meropenem, chloramphenicol, chloramphenicol, trimethoprim, erythromycin, azithromycin, sulfisoxazole | IncHI2, IncHI2A, IncR |
| | **Eho-E2/GN1532** | aac(6')-Ib-cr, aac(6')-Ib3, aadA1, blaOXA-1, blaVIM-1, catB4, dfrA14, mph(A), qnrB1, qnrS1, sul1 | ciprofloxacin I/R, gentamicin, streptomycin, ampicillin, amoxicillin/clavulanic acid, cefoxitin, ceftriaxone, meropenem, chloramphenicol, trimethoprim, erythromycin, azithromycin, sulfisoxazole | IncHI2, IncHI2A, IncR |

| | | | | |
|---|---|---|---|---|
| OTHER SPECIES | **Cfr-12/GN1092** | aac(3)-Ia, aac(3)-IId, aac(6')-Ib-cr, aac(6')-Ib3, aac(6')-Il, aph(3')-Ia, ARR-3, blaCMY-39, blaOXA-1, blaTEM-1B, blaVIM-2, catB4, mph(A), qnrA1, qnrB13, sul1 | gentamicin, ciprofloxacin I/R, kanamycin, rifampicin, ampicillin, amoxicillin/clavulanic acid, cefoxitin, ceftriaxone, meropenem, chloramphenicol, erythromycin, azithromycin, sulfisoxazole | IncHI2, IncHI2A, IncR |
| | **Cfr-13a/GN1762** | aac(6')-Ib-cr, aac(6')-Ib3, aadA1, blaCMY-34, blaVIM-1, dfrA14, mph(A), qnrB9, qnrS1, sul1 | ciprofloxacin I/R, gentamicin, streptomycin, ampicillin, amoxicillin/clavulanic acid, cefoxitin, ceftriaxone, meropenem, trimethoprim, erythromycin, azithromycin, sulfisoxazole | IncN, IncR |
| | **Kpn-13b/GN1763** | aac(6')-Ib-cr, aac(6')-Ib3, aadA1, blaSHV-11, blaVIM-1, dfrA14, fosA, mph(A), oqxA, oqxB, qnrS1, sul1 | ciprofloxacin I/R, gentamicin, streptomycin, ampicillin, amoxicillin/clavulanic acid, cefoxitin, ceftriaxone, meropenem, trimethoprim, fosfomycin, erythromycin, azithromycin, chloramphenicol, sulfisoxazole | IncN, IncR |
| | **Kpn-14/GN1761** | aac(3)-IIa, aac(6')-Il, aadA15, ant(3'')-Ia, aph(3')-Ia, aph(3')-VIb, aph(6)-Id, blaCMY-2, blaCTX-M-14b, blaSHV-1, blaTEM-1B, blaVIM-1, catA1, dfrA1, fosA, oqxA, oqxB, qnrS1, strA, sul1, tet(B) | gentamicin, streptomycin, kanamycin, ampicillin, amoxicillin/clavulanic acid, cefoxitin, ceftriaxone, meropenem, chloramphenicol, trimethoprim, fosfomycin, ciprofloxacin I/R, sulfisoxazole, tetracycline | IncA/C2, IncFIB(K), IncFII(K), IncHI1B, IncL/M(pMU407) |
| | **Eco-15/GN1765** | aac(6')-Ib-cr, aac(6')-Ib3, aadA1, aph(3')-XV, blaSHV-12, blaVIM-1, catB2, dfrA14, mph(A), sul1, tet(B) | ciprofloxacin I/R, gentamicin, streptomycin, kanamycin, ampicillin, ceftriaxone, amoxicillin/clavulanic acid, cefoxitin, meropenem, chloramphenicol, trimethoprim, erythromycin, azithromycin, sulfisoxazole, tetracycline | Col156, IncA/C, IncFII(29) |
| Other Ecl env isolates | **Eho-E3/GN1768** | aac(6')-Ib-cr, aac(6')-Ib3, aadA1, aadA16, aph(6)-Id, ARR-3, blaACC-1, blaACT-7, blaVIM-1, dfrA27, mph(A), qnrB6, strA, sul1, sul2, tet(A) | gentamicin, ciprofloxacin I/R, streptomycin, kanamycin, rifampicin, ampicillin, amoxicillin/clavulanic acid, cefoxitin, ceftriaxone, meropenem, trimethoprim, erythromycin, azithromycin, sulfisoxazole, tetracycline | IncHI2, IncHI2A, IncN, IncR, IncX5 |
| | **Eho-E4/GN1766** | aac(6')-Ib-cr, aac(6')-Ib3, aadA1, aadA16, aph(6)-Id, ARR-3, blaACC-1, blaACT-7, blaVIM-1, dfrA27, mph(A), qnrB6, strA, sul1, sul2, tet(A) | ciprofloxacin I/R, gentamicin, streptomycin, kanamycin, rifampicin, ampicillin, amoxicillin/clavulanic acid, cefoxitin, ceftriaxone, meropenem, trimethoprim, erythromycin, azithromycin, sulfisoxazole, tetracycline | IncHI2, IncHI2A, IncN, IncR, IncX5 |

**Table S4. Results of PlasmidFinder**

| Isolate ID | Plasmid | %Identity | %Overlap | HSP Length/Total Length | Contig | Start | End | Accession |
|---|---|---|---|---|---|---|---|---|
| **Eho-1/GN1534** | IncFIB(pEhoA) | 100 | 100 | 560/560 | 2 | 35915 | 36474 | CP001919 |
| **Eho-1/GN1534** | IncFII(pEhoA) | 100 | 100 | 747/747 | 2 | 3 | 749 | CP001919 |
| **Eho-1/GN1534** | IncQ1 | 100 | 81.28 | 647/796 | 3 | 647 | 1 | M28829.1 |
| **Eho-2/GN719** | IncFII(Yp) | 99.52 | 90.87 | 209/230 | 2 | 105039 | 105247 | CP000670 |
| **Eho-2/GN719** | IncN | 99.61 | 100 | 514/514 | 3 | 39 | 552 | AY046276 |
| **Eho-2/GN719** | IncR | 100 | 100 | 251/251 | 4 | 9889 | 10139 | DQ449578 |
| **Eho-3/GN738** | IncHI2 | 100 | 100 | 327/327 | 2 | 15542 | 15868 | BX664015 |
| **Eho-3/GN738** | IncN | 99.61 | 100 | 514/514 | 4 | 39 | 552 | AY046276 |
| **Eho-4/GN825** | Col156 | 98.7 | 100 | 154/154 | 3 | 20270 | 20423 | 009781 |
| **Eho-4/GN825** | IncFII(29) | 99.61 | 100 | 259/259 | 3 | 45041 | 45299 | CP003035 |
| **Eho-4/GN825** | IncFII(Yp) | 99.52 | 90.87 | 209/230 | 4 | 105039 | 105247 | CP000670 |
| **Eho-4/GN825** | IncHI2 | 100 | 100 | 327/327 | 2 | 329621 | 329295 | BX664015 |
| **Eho-4/GN825** | IncHI2A | 100 | 100 | 630/630 | 2 | 211 | 840 | BX664015 |
| **Eho-4/GN825** | IncN | 99.61 | 100 | 514/514 | 5 | 39 | 552 | AY046276 |
| **Eho-4/GN825** | IncR | 100 | 100 | 251/251 | 6 | 25273 | 25523 | DQ449578 |
| **Eho-5/GN882** | IncHI2 | 100 | 100 | 327/327 | 2 | 295831 | 295505 | BX664015 |
| **Eho-5/GN882** | IncHI2A | 100 | 100 | 630/630 | 2 | 211 | 840 | BX664015 |
| **Eho-5/GN882** | IncN | 99.61 | 100 | 514/514 | 2 | 113678 | 114191 | AY046276 |
| **Eho-5/GN882** | IncR | 100 | 100 | 251/251 | 3 | 22185 | 21935 | DQ449578 |
| **Eho-6/GN1550** | IncHI2 | 100 | 100 | 327/327 | 2 | 295128 | 294802 | BX664015 |
| **Eho-6/GN1550** | IncHI2A | 100 | 100 | 630/630 | 2 | 211 | 840 | BX664015 |
| **Eho-6/GN1550** | IncR | 100 | 100 | 251/251 | 4 | 13123 | 13373 | DQ449578 |
| **Eho-7/GN1157** | IncHI2 | 100 | 100 | 327/327 | 2 | 289263 | 288937 | BX664015 |
| **Eho-7/GN1157** | IncHI2A | 100 | 100 | 630/630 | 2 | 211 | 840 | BX664015 |
| **Eho-7/GN1157** | IncR | 100 | 100 | 251/251 | 6 | 24568 | 24818 | DQ449578 |
| **Eho-8/GN1318** | IncHI2 | 100 | 100 | 327/327 | 2 | 292818 | 292492 | BX664015 |
| **Eho-8/GN1318** | IncHI2A | 100 | 100 | 630/630 | 2 | 211 | 840 | BX664015 |
| **Eho-8/GN1318** | IncR | 100 | 100 | 251/251 | 5 | 3155 | 3405 | DQ449578 |
| **Eho-9/GN1494** | IncHI2 | 100 | 100 | 327/327 | 2 | 292818 | 292492 | BX664015 |
| **Eho-9/GN1494** | IncHI2A | 100 | 100 | 630/630 | 2 | 211 | 840 | BX664015 |
| **Eho-9/GN1494** | IncR | 100 | 100 | 251/251 | 6 | 20006 | 19756 | DQ449578 |

| | | | | | | | | |
|---|---|---|---|---|---|---|---|---|
| **Eho-10/GN1549** | IncHI2 | 100 | 100 | 327/327 | 3 | 272695 | 272369 | BX664015 |
| **Eho-10/GN1549** | IncHI2A | 100 | 100 | 630/630 | 3 | 211 | 840 | BX664015 |
| **Eho-10/GN1549** | IncR | 100 | 100 | 251/251 | 9 | 29830 | 30080 | DQ449578 |
| **Eho-11/GN1555** | IncHI2 | 100 | 100 | 327/327 | 2 | 295128 | 294802 | BX664015 |
| **Eho-11/GN1555** | IncHI2A | 100 | 100 | 630/630 | 2 | 211 | 840 | BX664015 |
| **Eho-11/GN1555** | IncR | 100 | 100 | 251/251 | 5 | 29644 | 29894 | DQ449578 |
| **Eho-E1/GN1533** | IncHI2 | 100 | 100 | 327/327 | 2 | 317217 | 316891 | BX664015 |
| **Eho-E1/GN1533** | IncHI2A | 100 | 100 | 630/630 | 2 | 211 | 840 | BX664015 |
| **Eho-E1/GN1533** | IncN | 99.81 | 100 | 514/514 | 5 | 39 | 552 | AY046276 |
| **Eho-E1/GN1533** | IncR | 100 | 100 | 251/251 | 4 | 11271 | 11521 | DQ449578 |
| **Eho-E1/GN1533** | IncX5 | 100 | 100 | 576/576 | 6 | 7364 | 6789 | MF062700.1 |
| **Eho-E2/GN1532** | IncHI2 | 100 | 100 | 327/327 | 1 | 3734347 | 3734673 | BX664015 |
| **Eho-E2/GN1532** | IncHI2A | 100 | 100 | 630/630 | 1 | 3719481 | 3718852 | BX664015 |
| **Eho-E2/GN1532** | IncR | 100 | 100 | 251/251 | 3 | 642 | 392 | DQ449578 |
| **Eho-E3/GN1768** | IncHI2 | 100 | 100 | 327/327 | 2 | 117227 | 116901 | BX664015 |
| **Eho-E3/GN1768** | IncHI2A | 99.21 | 100.16 | 631/630 | 2 | 132073 | 132701 | BX664015 |
| **Eho-E3/GN1768** | IncN | 98.84 | 100.39 | 516/514 | 4 | 5167 | 4653 | AY046276 |
| **Eho-E3/GN1768** | IncR | 99.6 | 100 | 251/251 | 3 | 72364 | 72614 | DQ449578 |
| **Eho-E3/GN1768** | IncX5 | 99.3 | 99.83 | 575/576 | 5 | 30014 | 29443 | MF062700.1 |
| **Eho-E4/GN1766** | IncHI2 | 99.69 | 100 | 327/327 | 2 | 76393 | 76718 | BX664015 |
| **Eho-E4/GN1766** | IncHI2A | 99.84 | 100 | 630/630 | 2 | 61543 | 60915 | BX664015 |
| **Eho-E4/GN1766** | IncN | 99.42 | 100 | 514/514 | 5 | 39 | 552 | AY046276 |
| **Eho-E4/GN1766** | IncR | 99.6 | 100 | 251/251 | 3 | 47022 | 47272 | DQ449578 |
| **Eho-E4/GN1766** | IncX5 | 99.65 | 99.65 | 574/576 | 6 | 44119 | 43548 | MF062700.1 |
| **Cfr-12/GN1092** | IncHI2 | 100 | 100 | 327/327 | 2 | 366162 | 365836 | BX664015 |
| **Cfr-12/GN1092** | IncHI2A | 100 | 100 | 630/630 | 2 | 211 | 840 | BX664015 |
| **Cfr-12/GN1092** | IncR | 100 | 100 | 251/251 | 4 | 26371 | 26121 | DQ449578 |
| **Cfr-13a/GN1762** | IncN | 99.61 | 100 | 514/514 | 3 | 39 | 552 | AY046276 |
| **Cfr-13a/GN1762** | IncR | 100 | 100 | 251/251 | 3 | 15001 | 14751 | DQ449578 |
| **Kpn-13b/GN1763** | IncN | 99.61 | 100 | 514/514 | 3 | 39 | 552 | AY046276 |
| **Kpn-13b/GN1763** | IncR | 100 | 100 | 251/251 | 3 | 16006 | 15756 | DQ449578 |
| **Kpn-14/GN1761** | IncA/C2 | 100 | 100 | 417/417 | 4 | 45 | 461 | JN157804 |
| **Kpn-14/GN1761** | IncFIB(K) | 98.93 | 100 | 560/560 | 3 | 859 | 300 | JN233704 |

| | | | | | | | | |
|---|---|---|---|---|---|---|---|---|
| **Kpn-14/GN1761** | IncFII(K) | 98.65 | 100 | 148/148 | 3 | 119262 | 119115 | CP000648 |
| **Kpn-14/GN1761** | IncHI1B | 99.47 | 100 | 570/570 | 2 | 655 | 86 | JN420336 |
| **Kpn-14/GN1761** | IncL/M(pMU407) | 99.55 | 90.66 | 670/739 | 5 | 72383 | 73052 | U27345 |
| **Eco-15/GN1765** | Col156 | 98.7 | 100 | 154/154 | 3 | 20275 | 20428 | 009781 |
| **Eco-15/GN1765** | IncA/C | 98.32 | 100 | 417/417 | 2 | 45 | 461 | FJ705807 |
| **Eco-15/GN1765** | IncFII(29) | 99.61 | 100 | 259/259 | 3 | 51867 | 52125 | CP003035 |

**Table S5. Summary of multilocus sequence typing results**

| Isolate ID | Scheme | Sequence Type | Locus 1 | Locus 2 | Locus 3 | Locus 4 | Locus 5 | Locus 6 | Locus 7 |
|---|---|---|---|---|---|---|---|---|---|
| **Eho-1/GN1534** | ecloacae | 92 | dnaA(52) | fusA(20) | gyrB(19) | leuS(44) | pyrG(45) | rplB(12) | rpoB(32) |
| **Eho-2/GN719** | ecloacae | 93 | dnaA(9) | fusA(4) | gyrB(14) | leuS(61) | pyrG(37) | rplB(4) | rpoB(9) |
| **Eho-3/GN738** | ecloacae | 93 | dnaA(9) | fusA(4) | gyrB(14) | leuS(61) | pyrG(37) | rplB(4) | rpoB(9) |
| **Eho-4/GN825** | ecloacae | 93 | dnaA(9) | fusA(4) | gyrB(14) | leuS(61) | pyrG(37) | rplB(4) | rpoB(9) |
| **Eho-5/GN882** | ecloacae | 93 | dnaA(9) | fusA(4) | gyrB(14) | leuS(61) | pyrG(37) | rplB(4) | rpoB(9) |
| **Eho-6/GN1550** | ecloacae | 269 | dnaA(95) | fusA(56) | gyrB(112) | leuS(111) | pyrG(104) | rplB(4) | rpoB(63) |
| **Eho-7/GN1157** | ecloacae | 269 | dnaA(95) | fusA(56) | gyrB(112) | leuS(111) | pyrG(104) | rplB(4) | rpoB(63) |
| **Eho-8/GN1318** | ecloacae | 269 | dnaA(95) | fusA(56) | gyrB(112) | leuS(111) | pyrG(104) | rplB(4) | rpoB(63) |
| **Eho-9/GN1494** | ecloacae | 269 | dnaA(95) | fusA(56) | gyrB(112) | leuS(111) | pyrG(104) | rplB(4) | rpoB(63) |
| **Eho-10/GN1549** | ecloacae | 269 | dnaA(95) | fusA(56) | gyrB(112) | leuS(111) | pyrG(104) | rplB(4) | rpoB(63) |
| **Eho-11/GN1555** | ecloacae | 269 | dnaA(95) | fusA(56) | gyrB(112) | leuS(111) | pyrG(104) | rplB(4) | rpoB(63) |
| **Eho-E1/GN1533** | ecloacae | 93 | dnaA(9) | fusA(4) | gyrB(14) | leuS(61) | pyrG(37) | rplB(4) | rpoB(9) |
| **Eho-E2/GN1532** | ecloacae | 269 | dnaA(95) | fusA(56) | gyrB(112) | leuS(111) | pyrG(104) | rplB(4) | rpoB(63) |
| **Eho-E3/GN1768** | ecloacae | 93 | dnaA(9) | fusA(4) | gyrB(14) | leuS(61) | pyrG(37) | rplB(4) | rpoB(9) |
| **Eho-E4/GN1766** | ecloacae | 93 | dnaA(9) | fusA(4) | gyrB(14) | leuS(61) | pyrG(37) | rplB(4) | rpoB(9) |
| **Cfr-12/GN1092** | cfreundii | 129 | aspC(7) | clpX(6) | fadD(76) | mdh(45) | arcA(3) | dnaG(45) | lysP(63) |
| **Cfr-13a/GN1762** | cfreundii | not assigned | aspC(9) | clpX(~11) | fadD(4) | mdh(95) | arcA(59) | dnaG(~66) | lysP(106) |
| **Kpn-13b/GN1763** | kpneumoniae | 17 | gapA(2) | infB(1) | mdh(1) | pgi(1) | phoE(4) | rpoB(4) | tonB(4) |
| **Kpn-14/GN1761** | kpneumoniae | 376 | gapA(2) | infB(6) | mdh(1) | pgi(3) | phoE(8) | rpoB(1) | tonB(27) |
| **Eco-15/GN1765** | ecoli | 131 | adk(53) | fumC(40) | gyrB(47) | icd(13) | mdh(36) | purA(28) | recA(29) |

## Table S6. Settings

| Key | Value |
|---|---|
| **command_line** | /ComputeShed/Applications/anaconda2/envs/staramr/bin/staramr search -o out11 GN1092.fasta GN1157.fasta GN1318.fasta GN1494.fasta GN1532.fasta GN1533.fasta GN1534.fasta GN1549.fasta GN1550.fasta GN1555.fasta GN1761.fasta GN1762.fasta GN1763.fasta GN1765.fasta GN1766.fasta GN1768.fasta GN719.fasta GN738.fasta GN825.fasta GN882.fasta |
| **version** | 0.6.1 |
| **start_time** | 2019-12-12 11:40:22 |
| **end_time** | 2019-12-12 11:40:45 |
| **total_minutes** | 0.38 |
| **resfinder_db_dir** | /ComputeShed/Applications/anaconda2/envs/staramr/lib/python3.7/site-packages/staramr/databases/data/dist/resfinder |
| **resfinder_db_url** | https://bitbucket.org/genomicepidemiology/resfinder_db.git |
| **resfinder_db_commit** | e8f1eb2585cd9610c4034a54ce7fc4f93aa95535 |
| **resfinder_db_date** | Mon, 16 Jul 2018 16:58 |
| **pointfinder_db_dir** | /ComputeShed/Applications/anaconda2/envs/staramr/lib/python3.7/site-packages/staramr/databases/data/dist/pointfinder |
| **pointfinder_db_url** | https://bitbucket.org/genomicepidemiology/pointfinder_db.git |
| **pointfinder_db_commit** | 8706a6363bb29e47e0e398c53043b037c24b99a7 |
| **pointfinder_db_date** | Wed, 04 Jul 2018 14:27 |
| **plasmidfinder_db_dir** | /ComputeShed/Applications/anaconda2/envs/staramr/lib/python3.7/site-packages/staramr/databases/data/dist/plasmidfinder |
| **plasmidfinder_db_url** | https://bitbucket.org/genomicepidemiology/plasmidfinder_db.git |
| **plasmidfinder_db_commit** | 81919954cbedaff39056610ab584ab4c06011ed8 |
| **plasmidfinder_db_date** | Tue, 20 Nov 2018 08:51 |
| **mlst_version** | 2.11 |
| **pointfinder_gene_drug_version** | 050218 |
| **resfinder_gene_drug_version** | 050218.1 |
